# Local niches explain coexistence in environmentally-distinct contact zones between Western Mediterranean vipers

**DOI:** 10.1038/s41598-023-48204-3

**Published:** 2023-11-30

**Authors:** Inês Freitas, Pedro Tarroso, Óscar Zuazo, Ricardo Zaldívar, Javier Álvarez, Manuel Meijide-Fuentes, Federico Meijide, Fernando Martínez-Freiría

**Affiliations:** 1 CIBIO, Centro de Investigação em Biodiversidade e Recursos Genéticos, InBIO Laboratório Associado, Universidade do Porto, Campus de Vairão, 4485-661 Vairão, Portugal; 2 BIOPOLIS Program in Genomics, Biodiversity and Land Planning, CIBIO, Campus de Vairão, 4485-661 Vairão, Portugal; 3 Departamento de Biologia, Faculdade de Ciências, Universidade do Porto, 4099-002 Porto, Portugal; 4 Santo Domingo de la Calzada, Spain; 5 Logroño, Spain; 6 Pradejón, Spain; 7 Golmayo, Soria, Spain; 8 Soria, Spain

**Keywords:** Ecology, Biogeography, Ecological modelling, Evolutionary ecology

## Abstract

Species’ ecological niches are frequently analysed to gain insights into how anthropogenic changes affect biodiversity. Coping with these changes often involves shifts in niche expression, which can disrupt local biotic interactions. Secondary contact zones, where competition and ecological segregation commonly occur, are ideal for studying the ecological factors influencing species’ niches. In this study, we investigated the effect of climate and landscape factors on the ecological niches of two viper species, *Vipera aspis* and *Vipera latastei*, across three contact zones in northern Iberia, characterized by varying levels of landscape alteration. Using niche overlap tests, ecological niche models and spatial analyses we observed local variation in the expression of the species’ niches across the three contact zones, resulting from the different abiotic and biotic conditions of each area. Rather than spatial niche segregation, we observed high niche overlap, suggesting niche convergence. A pattern of asymmetrical niche variation was identified in all contact zones, driven by species' climatic tolerances and the environmental conditions of each area. *V. aspis* generally exhibited a wider niche, except in the southernmost zone where the pure Mediterranean climate favored *V. latastei*. Human-induced landscape changes intensified niche asymmetry, by favoring the most generalist *V. aspis* over the specialist *V. latastei*, increasing habitat overlap, and likely competition. This study presents a comprehensive analysis of niche expression at range margins, anticipating a heightened impact of landscape changes in *V. latastei*. The methodological framework implemented here, and our findings, hold significant relevance for biodiversity management and conservation in human-impacted areas.

## Introduction

The distribution of a species is the expression of the complex interactions of abiotic and biotic factors that constrains population growth and limit its realized ecological niche^1^. These factors can vary considerably as response to natural (e.g. climate oscillations, topography) and/or anthropogenic changes (e.g. global warming, agriculture intensification)^2^. Species can cope with such changes through phenotypic plasticity and/or local adaptation, varying the local expression of their ecological niche^3,4^. However, not all species have the same capacity of response to environmental change. Generalist species (broad ecological niches) are more likely to exhibit high levels of plasticity and to be more resilient, while specialists (narrow ecological niches) tend to maintain habitat preferences and to be vulnerable to disturbance^4,5^. Under the homogenization and simplification of natural habitats that occurs with anthropogenic landscape change^6,7^, generalists are frequently favoured in detriment of specialist^8,9^. Yet, predicting the effects of anthropogenic landscape change on species and their interactions remains a challenging field of research.

Ecological niche-based models (ENM) correlate species occurrence with environmental variables, to infer the realized ecological niches of species and allow to make predictions of their potential distribution^10^. ENM transferability is the process of projecting a model to other temporal and geographical scenarios, assuming constant ecological niches, and it constitutes an important tool in biogeographic research^11,12^. However, ENMs overlook biotic interactions, despite their great importance on the spatial organisation of species^13,14^. Distinct approaches have been developed to indirectly include the effect of biotic interactions on model predictions^15,16^ but a standard method for analysing these interactions is still lacking.

Secondary contact zones provide an excellent system to understand how biotic and abiotic factors interact and shape the expression of ecological niches of co-existing species. They often correspond to areas of climatic transition where closely related species with parapatric distributions meet^17,18^. Closely related species frequently share similar ecological requirements and thus, are likely to compete intensely in areas of overlap^19^. Consequently, ecological segregation can occur along three major axes, allowing resource partitioning and coexistence^20,21^: (1) spatial (habitat and/or microhabitat use); (2) temporal (activity patterns); and (3) trophic (diet). However, if resources are limited, competing species cannot coexist in equilibrium, and the most fitting species will ultimately outcompete and eliminate the other (competitive exclusion principle)^22^. ENMs have been commonly used to unveil patterns of habitat selection across contact zones, implicitly incorporating interspecific competition on model predictions^23,24^. However, how novel conditions resulting from anthropogenic changes in landscape can influence biotic interactions and affect species’ distributions in contact zones is rarely addressed.

**Figure 1:**
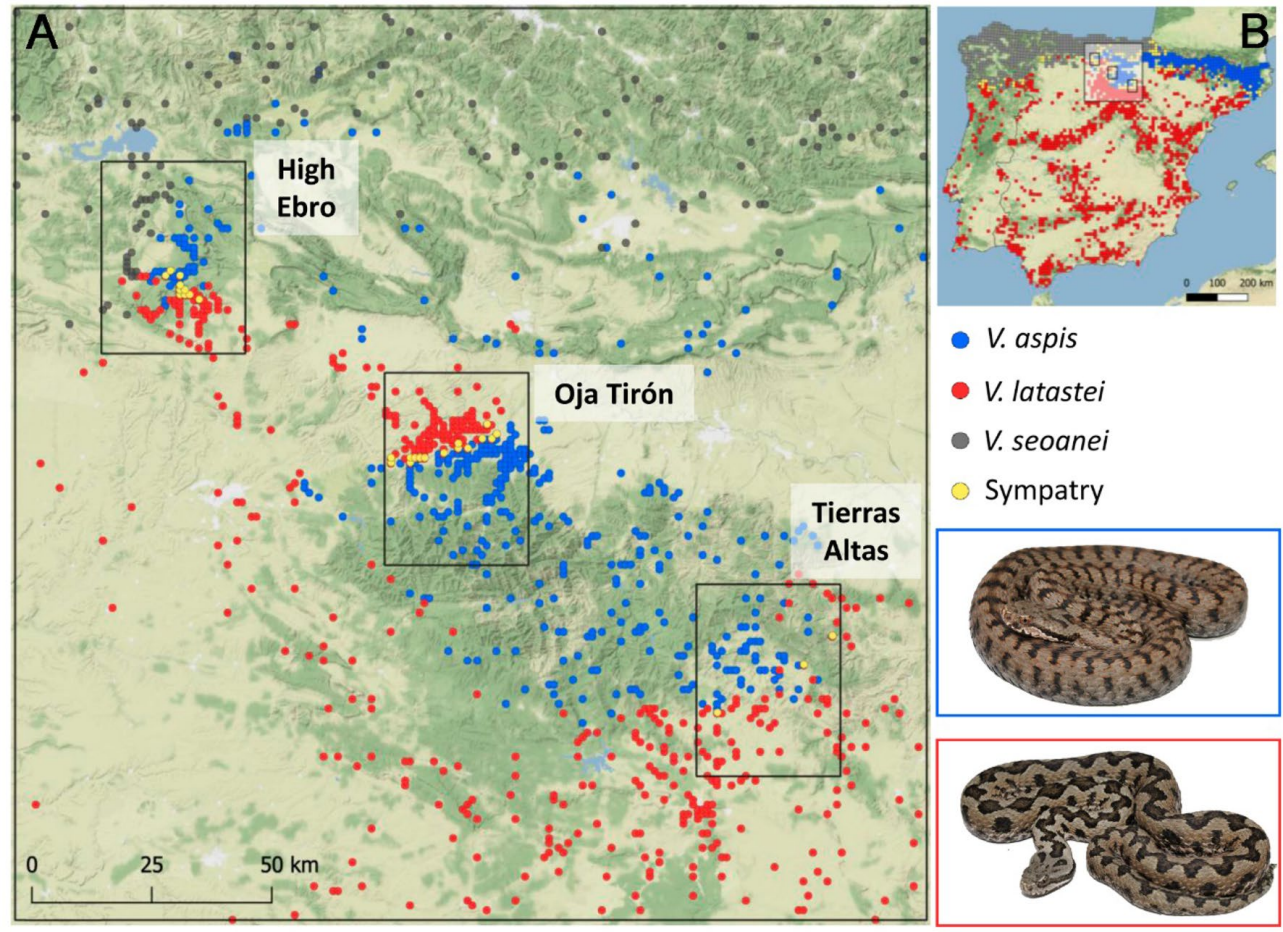
(**A**) Occurrence records of *V. aspis* (in blue), *V. latastei* (in red) and *V. seoanei* (in grey) in North Iberia and the three contact zones, High Ebro, Oja Tirón and Tierras Altas. (**B**) Distribution of the species in the Iberian Peninsula in a UTM 10 × 10 km grid and location of the study area. Pictures of *V. aspis* and *V. latastei* are shown inside blue and red boxes, respectively. Distribution data at UTM 10 × 10 km grid were obtained from Chamorro et al.^28^. Sympatry between species is represented in yellow. Background layer consists on a Stamen terrain map (http://maps.stamen.com/).

The Western Mediterranean vipers, *Vipera aspis* (Linnaeus, 1758) and *Vipera latastei* Boscá, 1878, constitute a unique system to investigate these questions. As ecthoterms, they have high thermal and hydric sensitivity and specific life-history traits (e.g. low dispersal and reproductive rate) that make them susceptible to range shifts and demographic alterations driven by environmental changes^25,26^. These species are phylogenetically closely-related and display parapatric distributions that overlap in areas of steep climatic transition in northern Iberia^25,27,28^. Studies using ENMs at distinct geographic scales suggested distinct ecological niches for these species, but with overlapping ecological requirements^16,24,25,28^. Remarkably, *V. aspis* has a wider and more generalist niche than *V. latastei*, which in the Iberian Peninsula is likely constrained by interspecific competition in contact zones^16,28^. In north-central Spain, both species are found in sympatry in three contact zones, characterized by different levels of anthropogenic landscape transformation (Fig. 1). Patterns of habitat selection at distinct scales, niche segregation (spatial and temporal) and hybridization were investigated in detail for the contact zone with lower human impact^16,24,29,30^. However, studies on the ecological factors allowing coexistence are still lacking for the other two contact zones.

Here, we develop a comparative framework to investigate how environmental factors (climate and landscape) shape the species’ distributions and affect the expression of their ecological niches in contact zones. For this purpose, we take advantage of a system that includes two phylogenetically closely-related species, *V. aspis* and *V. latastei*, with similar ecological requirements that are distributed across three a priori environmentally distinct contact zones. We rely on niche overlap tests, ENMs and spatial analyses to assess spatial patterns of niche variation within and across these contact zones, and in relation to a larger area including the three contact zones, where species distributions are expected to be less influenced by competitive interactions. Specifically, we address the following objectives: (1) to characterize and compare the environmental conditions of the contact zones and the species’ ecological niches; (2) to quantify the relative importance of climatic and landscape factors in the distribution of species across contact zones; and (3) to investigate how environmental factors affect the extent and location of the sympatric areas. Our hypothesis are: (1) contact zones are environmentally distinct and the expression of the species’ niches varies in accordance with the climatic and habitat variability of each area. Therefore, we expect that ENMs will have low transferability across areas; (2) since *V. aspis* and *V. latastei* share similar ecological requirements and are likely to compete in contact zones, spatial niche segregation is expected to occur. As a result, niche overlap between species will be less pronounced in the contact zones than in North Iberia, and ENMs predictions for these areas will underestimate species distribution in the larger area; and (3) the more generalist character of *V. aspis* will provide an ecological advantage over *V. latastei* in contact zones with human-disturbed landscapes. Therefore, we predict an asymmetrical niche reduction and stricter ecological preferences for *V. latastei*.

## Results

### Environmental variability of contact zones and species niches

Analyses on the environmental variability of contact zones show that they are climatically distinct, comparisons were statistically significant with low overlap values, but similar at the landscape level, comparisons were not significant and overlap index (OI) was close to maximum (Table 1).

**Table 1:** Pairwise comparisons of the environmental variability across contact zones (HE—High Ebro, OT—Oja-Tirón and TA—Tierras Altas) and ecological niches for both species (VAS—*V. aspis*, VLA—*V. latastei*) and across north Iberia (NIB) and the three contact zones.

Comparisons		Climatic					Landcover				
		Volumes (x/y)	Intersection	K	OI	pc1/pc2/pc3	Volumes (x/y)	Intersection	K	OI	pc1/pc2/pc3
Environmental variability	HE versus OT	4.545/3.812	0.265	0.065*	0.070*	0.373/0.268/0.220	35.245/70.861	34.568	0.644*	0.974	0.542/0.363/0.080
	HE versus TA	4.518/1.665	0.005	0.002*	0.003*		35.285/45.452	33.163	0.824*	0.941	
	OT versus TA	3.812/1.678	0.710	0.261*	0.426*		70.802/45.472	43.529	0.760*	0.963	
VAS	HE versus OT	2.185/1.598	0.083	0.045*	0.053*		23.883/68.978	21.245	0.463*	0.901	
	OT versus TA	1.609/1.034	0.262	0.200*	0.255*		69.728/36.392	34.318	0.658*	0.955	
	HE versus TA	2.185/1.039	0.003	0.002*	0.003*		23.966/36.421	17.865	0.583*	0.737*	
VLA	HE versus OT	1.293/0.339	0.007	0.009*	0.022*		21.029/17.369	7.648	0.396*	0.437*	
	OT versus TA	0.336/1.536	0.012	0.015*	0.041*		17.453/36.020	12.481	0.468*	0.722*	
	HE versus TA	1.300/1.536	0.000	0.000*	0.000*		20.926/35.966	16.894	0.597*	0.812	
NIB	VAS versus VLA	22.677/15.517	11.451	0.601	0.739*		67.726/51.059	47.474	0.803*	0.934	
HE	VAS versus VLA	2.197/1.288	0.983	0.560*	0.754*		23.956/20.993	16.906	0.762	0.810	
OT	VAS versus VLA	1.600/0.337	0.253	0.258*	0.741*		69.836/17.407	16.937	0.393*	0.977	
TA	VAS versus VLA	1.035/1.540	0.885	0.689	0.855		36.505/35.931	26.838	0.738	0.742*	

Niche volumes of the first (x) and second (y) groups in comparison, niche volumes intersection, Sorensen (K) and overlap (OI) indexes of niche overlap and contribution of the first three components of climatic and landcover PCAs are provided. Significant values of K and OI (*p* < 0.5) are signalled with *.

**Figure 2:**
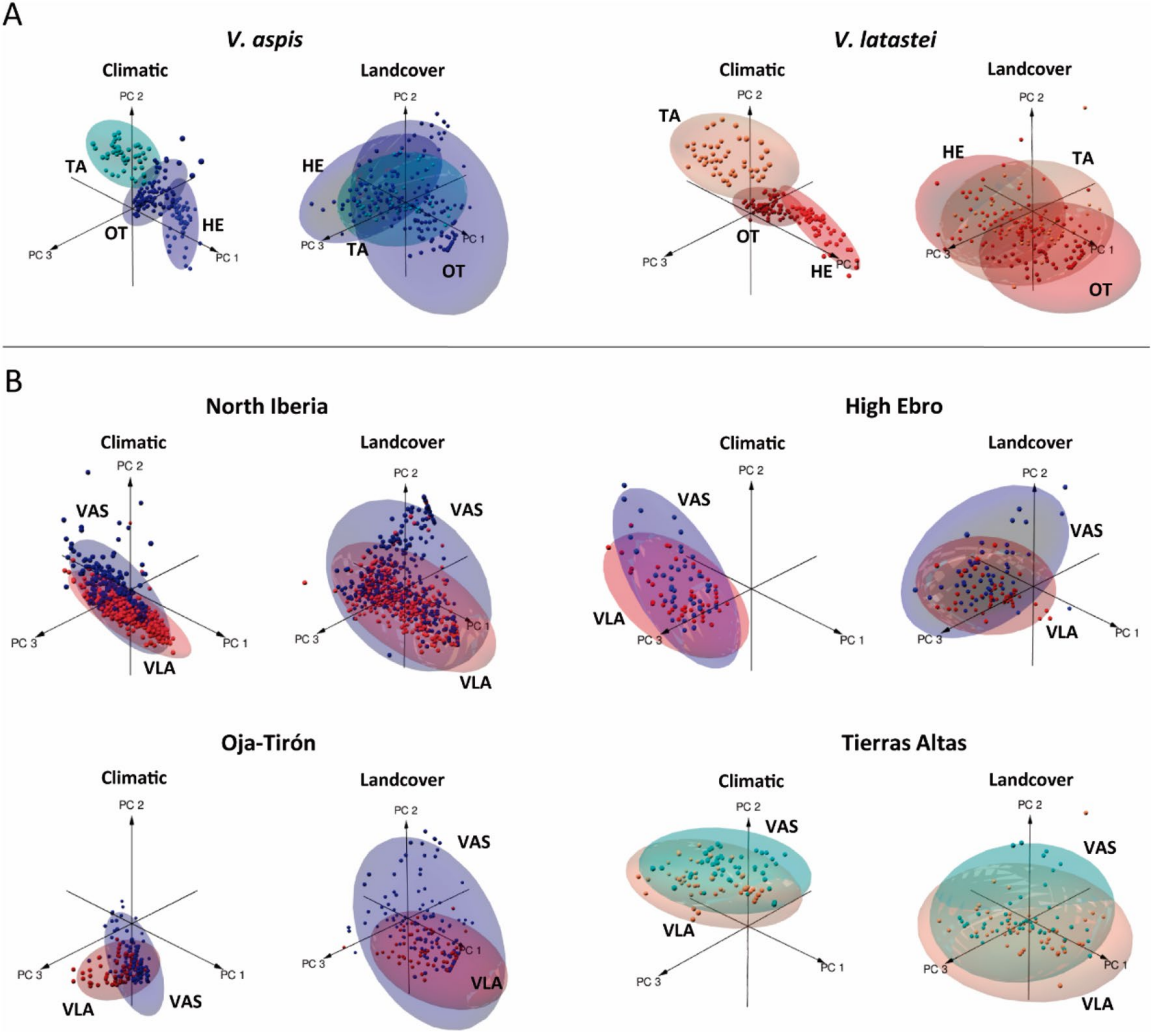
(**A**) Delimitation of the climatic and landcover niches of *V. aspis* and *V. latastei* across contact zones considering the three first components of the principal component analyses (PCA). (**B**) Niche intersection of *V. aspis* and *V. latastei* in North Iberia and the three contact zones, High Ebro, Oja-Tirón and Tierras Altas. Ellipses represent a confidence interval of 95%. *V. aspis* is coded in blue and *V. latastei* in red. Contact zones are represented with different shades of these colours.

Concerning the species’ climatic niches across contact zones, niche overlap was low (OI < 0.255; Table 1, Fig. 2A). When contrasting the volumes of the species’ niches with the climatic variability of the contact zones, the *V. latastei*’s niche includes almost all the climatic variability found in Tierras Altas (Table 1). Whereas, in Oja-Tirón and the High Ebro both species are using less than half of the climatic variability of these areas (Table 1). Niche comparisons based on landcover variables were statistically significant for most cases but with moderate to high overlap values (0.437 < OI < 0.955; Table 1, Fig. 2A). *V. aspis* has a broader landcover niche in Oja-Tirón, followed by Tierras Altas and the High Ebro. *V. latastei* has a wider niche in Tierras Altas and smaller in Oja-Tirón (Table 1, Fig. 2A).

Comparisons between species show that climatic niche overlap in North Iberia was high (OI = 0.739) and statistically significant, with *V. aspis* having the largest climatic niche (Table 1, Fig. 2B). In the contact zones, climatic niche overlap was high (0.741 < OI < 0.855) and statistically significant (except in Tierras Altas; Table 1). In the High Ebro and Oja-Tirón, the niche of *V. aspis* is respectively two and five times the size of *V. latastei*’ niche; in Tierras-Altas, *V. aspis’* niche is almost completely included within *V. latastei*’ niche (Table 1, Fig. 2B). Regarding the niches estimated with landcover variables, six out of the 12 comparisons done were not statistically significant, niche overlap was moderate to high for all comparisons (0.437 < OI < 0.977) (Table 1, Fig. 2B). *V. aspis* has a larger landcover niche in all areas, but differences in the niche ’sizes are only pronounced in Oja Tirón where *V. aspis*’ niche is four times the size of *V. latastei*’s niche (Table 1, Fig. 2B).

### Importance of climatic and habitat variables in the species’ distributions

Models’ performance in the training areas was variable among species and study areas (Supplementary Table S3). Climatic models and climatic+landcover models show high TSS (True Skill Statistics) values (TSS > 0.7), while landcover models have lower performance (0.1 < TSS < 0.5 for all models, except for *V. latastei*’ model in Oja-Tirón). Model replicates are consistent and standard deviation values are low for all cases (Supplementary Fig. S1).

**Figure 3:**
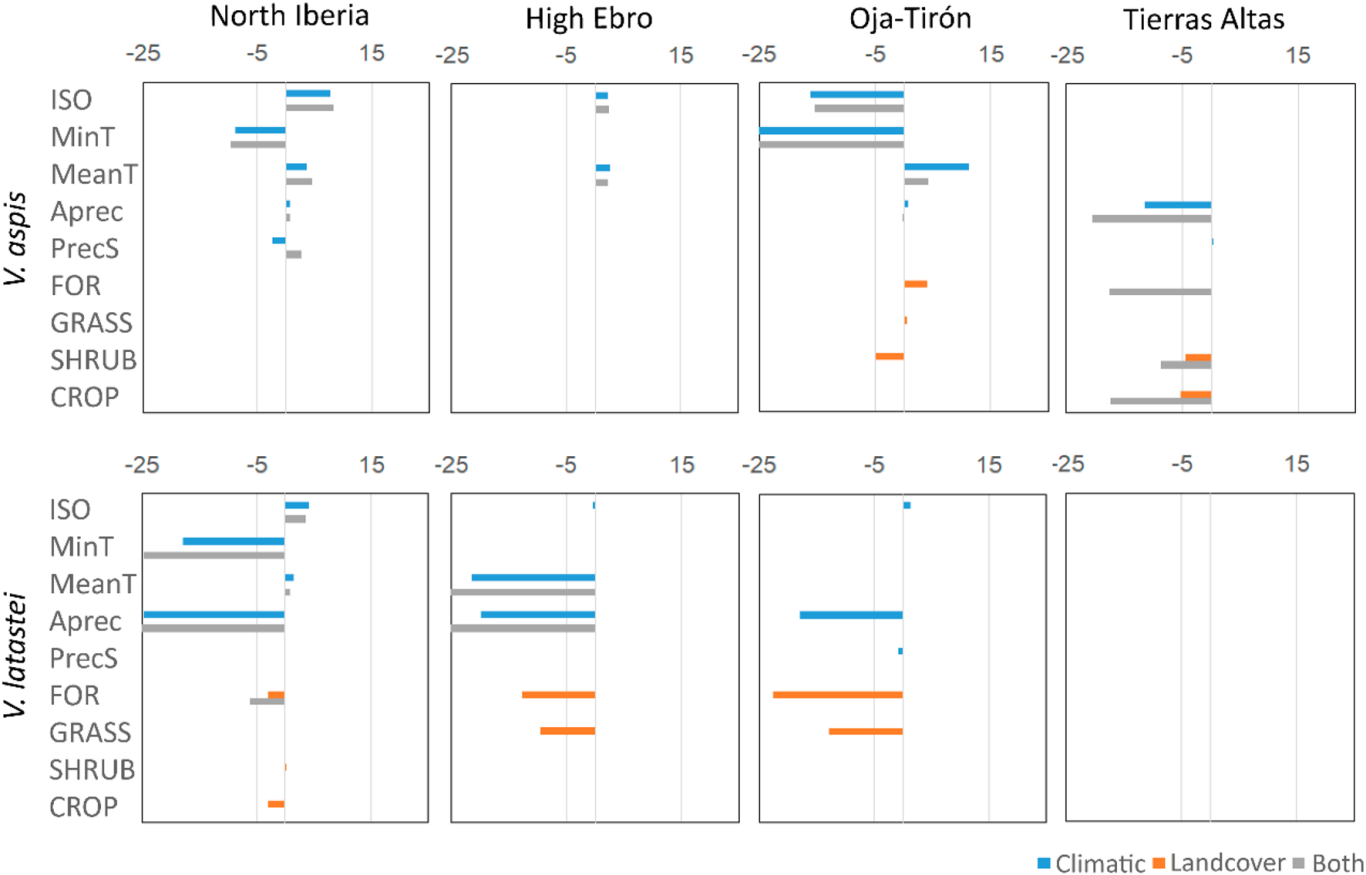
Average coefficients for the most important variables in ENMs developed for *V. aspis* (VAS) and *V. latastei* (VLA) in each area (North Iberia—NIB, High Ebro—HE, Oja-Tirón—OT, Tierras Altas—TA), for models developed with climate, landcover or both types of variables. See Supplementary Text S3 for further details on models and environmental correlates.

**Figure 4:**
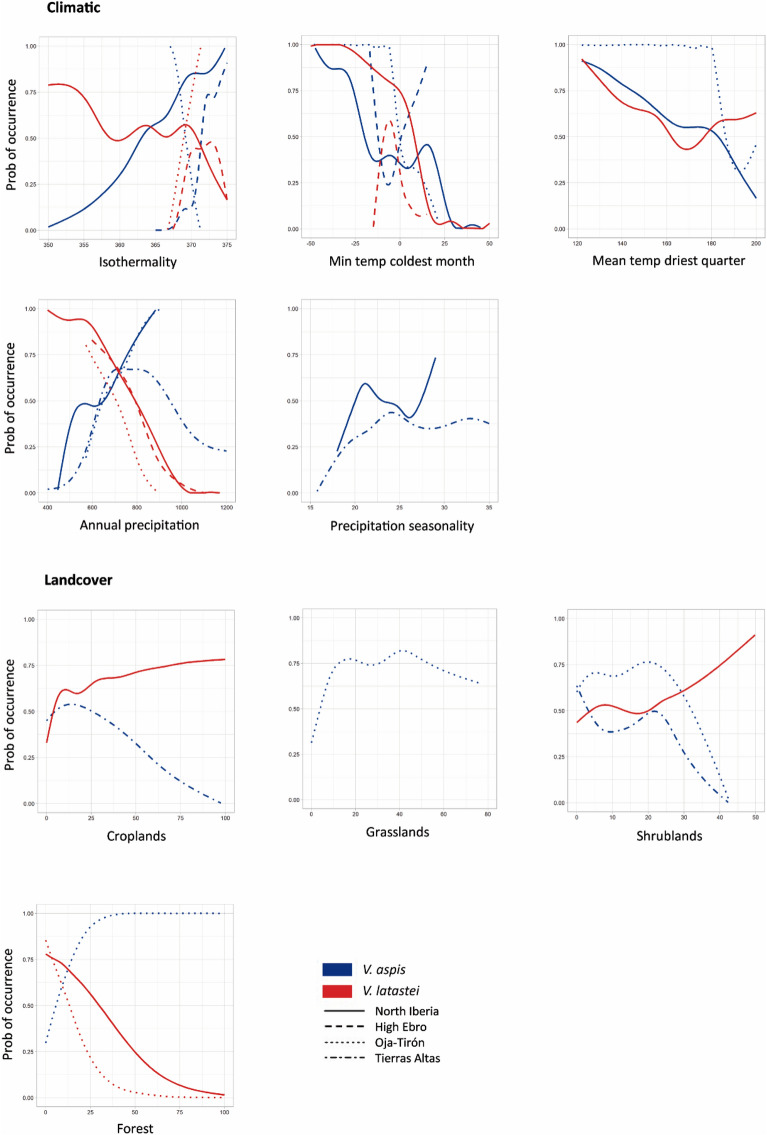
Response curves for the most important climatic and landcover variables explaining species’ distributions in the ENMs. Species are depicted with different colours (*V. aspis* coded in blue, and *V. latastei* in red), and contact zones are represented with different line types.

Environmental correlates and response curve profiles revealed distinct patterns of climatic and habitat selection across species and areas (Figs. 3 and 4). In North Iberia, the species distributions are influenced by the same climatic variables, but landcover correlates are only meaningful for *V. latastei* (Fig. 3; Supplementary Text S3). Response curves for these variables show that *V. aspis* prefers colder and more humid habitats, while *V. latastei* occurs in warmer and drier habitats with shrub vegetation and low forest cover (Fig. 4).

Across the three contact zones, different environmental correlates and contrasting responses stand out for almost all variables when compared to North Iberia (Figs. 3, 4; Supplementary Text S3). The distribution of *V. aspis* correlates with temperature (in the High Ebro), precipitation (Tierras Altas) or both climatic variables (Oja-Tirón) in the contact zones. Different landcover variables are also relevant for the distribution of this species in Oja-Tirón and Tierras Altas. Response curves for the common variables between North Iberia and contact zones show contrasting patterns of habitat selection in North Iberia, the High Ebro and Oja-Tirón in two temperature related variables (isothermality and minimum temperature of the coldest month), with the species selecting warmer habitats in the latter contact zone. For *V. latastei*, both temperature and precipitation variables are meaningful for the species distribution in the High Ebro and Oja-Tirón, showing similar response curves across areas, while landcover variables (forest cover) is only relevant in Oja-Tirón. Nonetheless, no climatic nor landcover variables stand as important for the species distribution in Tierras Altas.

Overall, climatic and landcover correlates are distinct between species across all areas, with species also selecting different habitats, as reflected by the opposite responses for most of the common variables (Fig. 3). For instance, in the High Ebro, *V. aspis* selects warmer areas while *V. latastei* prefers colder and drier habitats; and in Oja-Tirón, *V. aspis* selects cold and humid areas occupied by forests and *V. latastei* is present in warmer and drier environments, avoiding forests (Figs. 3 and 4). Model transferability between contact zones showed low TSS values (0.1 < TSS < 0.5) and deviations from Miller's intercept and slope calibrated values (Supplementary Table S3). Similarly, models trained in North Iberia and projected to the contact zones showed low ability to predict species distributions in these areas, with a consistent overestimation (Miller’s interception above 0) of presence probability (Supplementary Table S3).

### Spatial patterns of sympatry across contact zones

**Figure 5:**
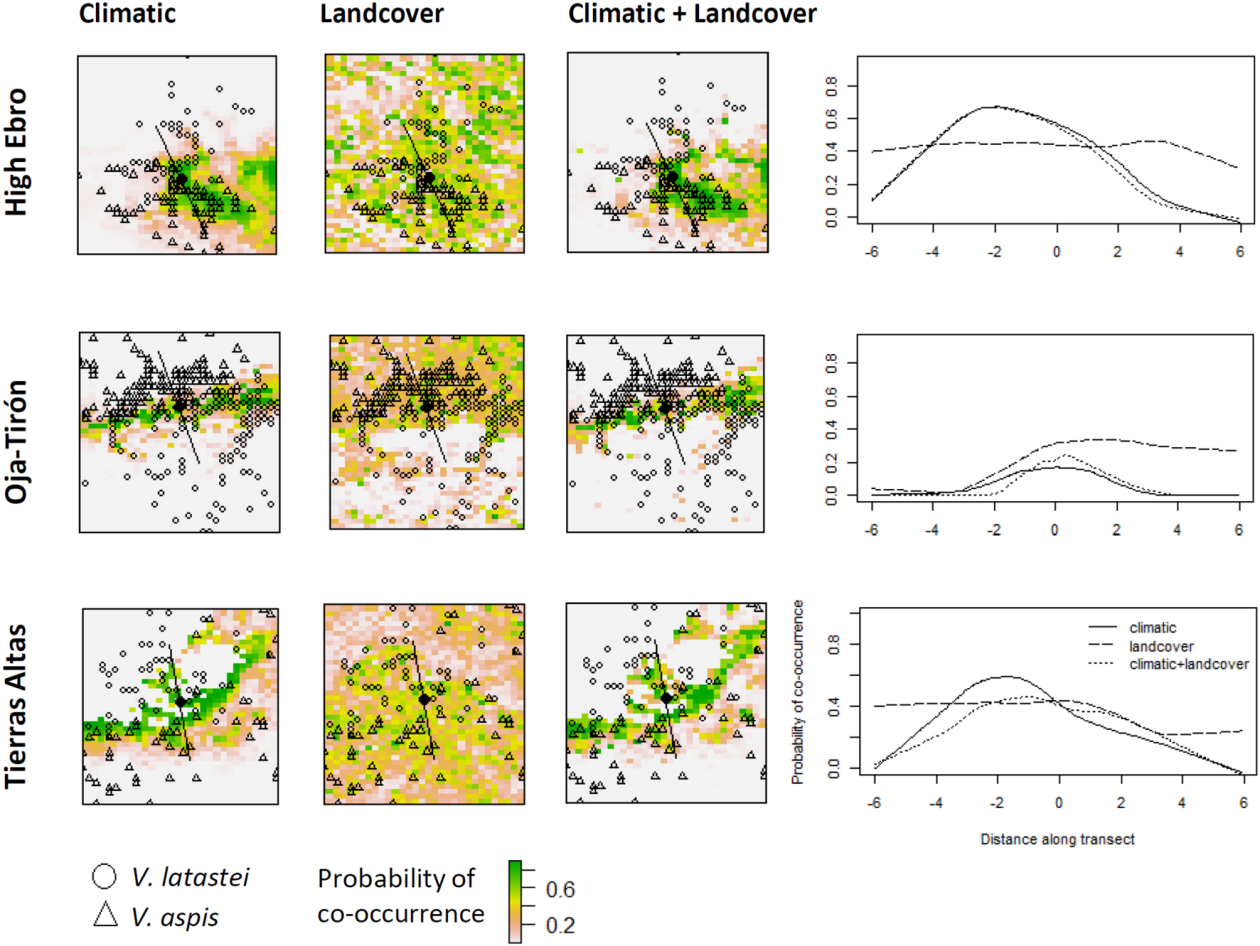
On the left, sympatry maps generated for each contact zone by the intersection of average probability maps obtained from the climatic, landcover and climatic+landcover models and a transect set perpendicularly to the contact between species. On the right, plot representing the probability of co-occurrence (obtained from the sympatry maps) along the transect.

Regarding the climatic and climatic+landcover sympatry maps, curves representing the probability of co-occurrence of both species along the transects show the existence of probable sympatric areas located between the distribution of the species for the three contact zones. For Oja-Tirón, however, this curve is less pronounced reaching low maximum values of co-occurrence probability. For the sympatry landcover maps, curves are flat for the High-Ebro and Tierras-Altas where the probable areas of sympatry are instead dispersed across the contact zones. For Oja-Tirón the models predict extensive areas of co-occurrence in the northern half of the contact zone, where only *V. latastei* occurs (Fig. 5).

## Discussion

### Local niches across contact zones

Our study reveals low overlap in the climatic niches of both species across the contact zones, but high overlap on the niches derived with landcover variables (Table 1), meaning that species tend to select areas with different climatic conditions but are mostly present in the same habitat types across contact zones.This pattern is consistent with the results of environmental variability for the three contact zones. Each contact zone has particular climatic conditions, while sharing a similar landscape composition (Supplementary Table S1, Supplementary Text S1). Contact zones vary structurally in terms of landscape (in abundance and spatial distribution of the main habitat types, Supplementary Table S1, Supplementary Text S1), but they are represented by the same four landcover layers, resulting in similar degrees of landscape variability (Table 1). Because the results of niche overlap across areas mirror the results of environmental variability of contact zones, there are strong indications that local environmental variation is driving the reported pattern of niche variation across areas in these species. This is that the species’ niches are much wider than their local niches as species are only able to fulfil restricted parts of their niche in each contact zone due to the different abiotic conditions^31^.

To evaluate if the observed niche differences between areas are exclusively the result of environmental availability we compared the niche of the species in each contact zone in relation to their niche in North Iberia, which includes the climatic and landcover variability of the other areas (“partial niche filling vs true niche shift” hypothesis)^31^. We found that different factors limit the distribution of the species in North Iberia and the three contact zones, as models exhibited distinct environmental correlates and projections fail to predict the distribution of the species outside training areas (Fig. 3, Supplementary Fig. S1). Moreover, response curves for the common important climatic and landcover variables recover distinct, and in some cases opposing, patterns of habitat selection across areas, which is particularly evident for *V. aspis* (e.g. isothermality, minimum temperature of the coldest month; Fig. 3). On the other hand, *V. latastei* generally maintains habitat preferences in all areas (Fig. 3), consistently avoiding for instance humid and canopied areas. Altogether, these findings indicate that the different abiotic conditions found in the contact zones explain in part the local variation on the expression of the ecological niches, but the opposing patterns of habitat selection across areas and the low model transferability from North Iberia to the contact zones, suggest the influence of other contributing factors. The particular biotic conditions of each area (such as the extent of interspecific competition) can most likely, influence habitat selection patterns and contribute to the local niche differences observed among areas, as reported in other studies^32,33^.

Overall, these results are in line with our initial hypothesis of distinct ecological niches being expressed locally due to variation in local abiotic and biotic conditions. Notably, this capacity to express local niches seems to be different for *V. aspis* and *V. latastei*, as evidenced by the contrasting patterns of habitat selection across areas obtained for *V. aspis* and the general tendency to maintain habitat preferences observed for *V. latastei*. There are important differences on the climatic tolerances of these species that can explain this pattern. Unlike *V. latastei*, *V. aspis* is not physiologically limited to Mediterranean conditions being also present in the Atlantic-influenced region^25,28^. The wider ecological niche of this species likely allows it to cope with a different range of conditions in the contact zones and more plasticity in habitat preferences across areas.

### Reduced spatial segregation across contact zones

Our study recovers extensive climatic and habitat niche overlap between *V. aspis* and *V. latastei* in the three contact zones and in North Iberia, an expected pattern of phylogenetic niche conservatism^19^, already reported at distinct scales for these species^16,25^. However, contrary to our initial expectations, the extent of niche overlap does not differ much from the contact zones to North Iberia. This may imply that interspecific competition is not a stronger force driving niche segregation in these areas more than in the wider area of contact. In fact, climatic niche overlap is lower in North Iberia than in the three contact zones, where species’ niches probably do not segregate their niches but instead converge to adapt to the same local conditions, resulting in similar local niches for both species^34,35^. A recent study comparing allopatric and sympatric populations of these species in the High Ebro found, as well, no segregation in habitat use in the sympatric area of this contact zone^16^. Remarkably, local adaptation to similar selective pressures has been previously reported as a factor explaining morphological convergence of both species across contact zones^27^.

Despite having high niche overlap and overall similar ecological requirements in the contact zones and in North Iberia, these species display important differences in the environmental correlates as expected from major allopatric distributions. Response curves showed contrasting responses for temperature and precipitation related variables and forest cover in Oja-Tirón (Fig. 4), indicating a general tendency for *V. aspis* to select more humid and colder habitats in more canopied areas than *V*. *latastei.* Consistently, studies conducted at the regional scale^25,28^ and at the local scale^16,24^ describe similar patterns of habitat selection for these species. Yet, differences in the species ecological preferences observed in contact zones and at the regional scale, are most likely attributed to past competitive pressures during coevolution (“ghost of competition past”)^36,37^, reflecting a natural segregation in habitat preferences due to diversification of physiological tolerances, and not an indication of niche segregation associated to intense competition in these areas^38,39^.

Besides spatial segregation, other mechanisms have been suggested to minimize interspecific competition and allow stable coexistence in contact zones^40–43^. In the High Ebro, previous studies found segregation in the activity patterns of adult males of both species, but no segregation in the trophic axis^29^. This may also be the case for the other contact zones between *V. aspis* and *V. latastei*. Further studies comparing activity patterns and prey consumption of these species are needed to address if temporal and trophic niche segregation, respectively, are occurring in the remaining contact zones.

### Asymmetrical niche variation between contact zones

Our results show that *V. aspis* has a much wider climatic and habitat niche than *V. latastei* in the High Ebro, Oja-Tirón and North Iberia, but not in Tierras Altas, where the climatic niche of *V. aspis* is smaller and the habitat niches are the same size for both species (Table 1; Fig. 2). A broader climatic niche and higher tolerance to cool environments has been indicated for *V. aspis* at the regional scale^28^, suggesting possible ecological advantage over *V. latastei* when they are in contact and a capacity to inhabit a wider range of environments in the contact zones.

In the High Ebro, asymmetrical niche reduction and stricter ecological preferences were detected for sympatric populations of *V. latastei* in relation to allopatric populations*,* likely as a result of the competitive pressure imposed by *V. aspis*, but not the opposite^16^. In our study, we observed the same pattern of asymmetrical niche variation for these species in this contact zone, although niche volumes did not vary considerably from the ones obtained for the species in North Iberia (Table 1; Fig. 2). This indicates that competition is probably weak and has a minor influence in the species’ niches in this contact zone, as already suggested by Scaramuzzi et al.^16^ The natural landscape of this area likely provides enough resources and microhabitats, reducing competitive interactions between species^26^. Indeed, previous studies found differences in the micro-habitat use and activity patterns of males of both species, which can be relaxing competitive interactions^29^. Therefore, the asymmetrical niche volumes observed in this contact zone likely reflect differences in these species eco-physiological tolerances, narrower for *V. latastei* and wider for *V. aspis,* than an effect of interspecific competition.

Remarkably, asymmetrical variation in the niches occurs in Oja-Tirón. In this contact zone, the niche of *V. latastei* is five-fold smaller than the niche of *V. aspis* (and much smaller than in the other contact zones; Table 1). While the climatic niches of *V. aspis* and *V. latastei* overlap less in Oja-Tirón than in the nearby contact zones (Table 1) and predicted area of sympatry is narrow (Fig. 5), the landcover niches of the two species overlap almost completely (Table 1). This suggests that in this contact zone, intensive agricultural practices have likely resulted in reduced habitat heterogeneity and overall habitat loss, ultimately leading to an extensive habitat overlap between species^6,44^. Land use is known to favour generalists over specialists, as they are able to cope better with habitat loss by adapting to the novel conditions or occupying other areas. This may translate into pronounced differences in the niche volumes of these species, with specialists as *V. latastei* experiencing a major niche reduction in relation to the generalists^9,45^. Therefore, it seems reasonable that the generalist character of *V. aspis* allows this species to inhabit a wider range of environmental conditions in Oja-Tirón, such as in the High Ebro, but landscape disturbance likely intensifies this pattern of asymmetrical variation in the niches. Moreover, the increased overlap between *V. aspis* and *V. latastei* might be exacerbating competition in this contact zone, resulting in niche displacement for *V. latastei* and its exclusion from environmental favourable areas by the better adapted *V. aspis*^46^.

Interestingly, in Tierras Altas, we detected an opposite pattern of asymmetrical niche variation from the other contact zones and North Iberia, with *V. aspis* showing a climatic niche reduction in relation to *V. latastei* (Table 1). Indeed, Chamorro et al.^28^ predicted *V. aspis* as a better competitor than *V. latastei* in transition areas between montane and Mediterranean zones, but not at the southern limit of its range where the pure Mediterranean conditions favour *V. latastei* instead. Therefore, we suggest that in the southernmost contact zone of North Iberia, the drier climate that characterizes this area favours *V. latastei* over *V. aspis*, resulting in the observed pattern of asymmetrical niche variation between species. For these peripheral populations of *V. aspis* that are at their environmental margin, fitness may already be much lower than in the centre of their distribution and interspecific competition with *V. latastei,* that is better adapted to local conditions, can as well impose additional constrains^47^.

Overall, environmental and physiological constrains coupled with competitive exclusion imposed by the better-adapted species seem to be the main factors limiting the species’ distributions in the three contact zones. A similar pattern has also been reported in the contact zones between *V. aspis* and *V. berus* in northern Italy^48^ and western France^49,50^, and at the regional scale for the three Iberian vipers^28^.

## Conclusions

This work tackles the distinct ecological drivers of species’ distributions in secondary contact zones and the effects of anthropogenic landscape change on their ecological niches. We found that local variation in the expression of the ecological niches across the three contact zones and species results from the particular abiotic and biotic settings in each area. Niche segregation was not evident in any contact zone, instead ecological convergence to local conditions likely resulted in the high niche overlap observed. Yet, an asymmetrical variation in the species’ niches was detected in all contact zones, particularly in Oja-Tirón, where anthropogenic landscape change likely favours the generalist *V. aspis* over *V. latastei* and increases competition. Our results suggest that *V. aspis* may have an ecological advantage over *V. latastei* in human-disturbed landscapes resulting in niche displacement for the outcompeted species. Nevertheless, the interplay between species distributions, biotic interactions and environmental conditions can rapidly shift in response to climate change^2^. Forecasted increases of aridity and temperature in the Iberian Peninsula may favour *V. latastei* over *V. aspis*, but further studies based on the species physiology are needed to address this in detail.

Overall, we offer a detailed analysis of niche expression at range margins, where competition with ecologically-similar species is prevalent. The methodological framework implemented in this study, along with its findings, carry significant implications for biodiversity management in human-impacted areas and for conservation efforts in response to the challenges posed by climate change.

## Material and methods

### Study area

We defined one large study area (hereafter North Iberia), located in north-central Spain with a total area of 36,100 km^2^, where both Western Mediterranean vipers stablish contact broadly (Fig. 1). Three smaller contact zones of 1,200 km^2^ were defined within North Iberia (Fig. 1): the High Ebro, Tierras Altas and Oja-Tirón, where local sympatry has been found^24,51,52^. These areas are characterized by distinct levels of human landscape disturbance: natural in the High Ebro, abandoned grazing fields in Tierras Altas and intensive culture fields of cereals in Oja-Tirón. Further details on the climatic and habitat characteristics of these areas can be consulted in Supplementary Text S1.

### Species occurrences and environmental factors

A total of 871 occurrence records (at 1 km^2^ resolution, UTM European 1950 datum zone 30N) were gathered from North Iberia, including 421 of *V. aspis* and 450 of *V. latastei* (Fig. 1). By contact zones, 112 records were restricted to the High Ebro (52 *V. aspis*, 60 *V. latastei*), 228 to Oja-Tirón (133 *V. aspis*, 95 *V. latastei*), and 100 to Tierras Altas (50 each species). A set of nine bioclimatic and four landcover variables, at a spatial resolution of 1 × 1 km and low correlated were selected for spatial analyses (Supplementary Table S1). Climatic and landcover variables were downloaded from CHELSA v1.2 (https://chelsa-climate.org/) and Copernicus (https://www.copernicus.eu/), respectively. Further details on occurrence records collection and environmental factors selection can be consulted in Supplementary Text S2.

### Environmental variability of contact zones and niche overlap analyses

To comparatively assess (1) the environmental variability of the three contact zones, (2) the extent of niche change across North Iberia and the three contact zones, and (3) the extent of niche segregation between species, we relied on a 3D hypervolume approach, using the “hypervolume” R package^53^.

We summarized the climatic and land cover variability of North Iberia by performing two Principal Component Analyses (PCA) separately. PCA scores were extracted for the species occurrences in each study area. Hypervolumes were built using a Silverman bandwidth estimator, a quantile threshold of 95%, and a set of 1000 random points to sample the kernel density. Pairwise niche overlap was quantified using Sørenson (K) and the Overlap indexes (OI)^25^. Both indexes range between zero (no overlap) and one (complete overlap). The Overlap Index (OI) relates the observed and maximum values of K and is preferred when niches present different sizes^54^. Randomization tests were performed based on 100 permutations to test whether the observed niche overlap is more different than the overlap between two simulated, randomly delimited niches^55^. All analyses were conducted using R version 4.0.5.

### Ecological-niche modelling

To quantify and compare the relative importance of climatic and landscape factors in the distribution of *V. aspis* and *V. latastei* across each area, we performed ENMs using Generalized Linear Models (GLM). This regression-based algorithm uses presence and absence data (true absences or pseudo-absences) and is less sensitive than other algorithms to geographically biased sampling^56^, as it is the case in the training area of North Iberia (see Supplementary Text S2). To implicitly account for interspecific competition between viper species, pseudo-absences were randomly selected within the range of the non-modelled species (delimited with a minimum convex polygon) using “dismo” package^57^. The number of pseudo-absences created equalled the number of records from the modelled species. In North Iberia and the High Ebro, we extended pseudo-absence data selection to the range of a third Iberian viper, *Vipera seoanei* (Fig. 1)*,* which is allopatrically distributed in relation to the two vipers of interest^24,30^. For that purpose, we considered 135 and 29 records of the species in North Iberia and the High Ebro, respectively, which were available from published data and fieldwork developed by the authors.

To disentangle the independent and joint effect of climate and landcover in the distribution of the species, ENMs were fitted using three sets of explanatory variables: (1) climate only; (2) land cover only; and (3) climate and land cover.

A total of 24 ENMs (i.e. 2 species × 3 sets of variables × 4 study areas) were performed. For each ENM, 30 replicates were run, randomly partitioning records in 80/20% for training/testing, and only the 10 best performing replicates were kept to derive an average model. Models’ performance in the training area was assessed using the true statistic skills (TSS) metric^58^.

To identify the most important factors related to species distributions, the logistic regression coefficients of each EGV were used as a measure of variable importance. The relation between the probability of species’ occurrence and the most important EGVs was estimated using the rasters from the average probability models and the EGVs.

To investigate how environmental correlates are spatially transferable, models trained in each contact zone were projected to the other two contact zones and to North Iberia. Potential extrapolation of the models was assessed with clamping masks using the MESS function (Multivariate Environmental Similarity Surfaces)^59^. TSS and the Miller’s^60^ calibration statistics were used to assess the performance of the model projections outside training areas. Both metrics were calculated using modEvA package^61^.

All analyses from data processing to model building were done with R version 4.0.

### Characterization of the sympatric areas

To investigate how the environmental factors can affect the extent and location of the sympatric areas across contact zones, the average probability models generated by ENMs were used to create sympatry maps and visualize the probability of co-occurrence along a transect set orthogonally to each contact zone. Average probability models for each pair of species were intersected using “fuzzySim” R package^62^ to obtain a continuous sympatry raster, representing the probability of co-occurrence across contact zones. A transect was set for each contact zone (placed at the center of the sympatric area and orthogonally to the average direction of the contact zone; Supplementary Table S2) to calculate the distance of each occurrence to the sympatric areas. The values of probability of co-occurrence were projected orthogonally to the transect and plotted against distance along transect to allow a better visualization of the sympatric areas.

### Supplementary Information

Supplementary Information.

## Data Availability

The datasets used and/or analysed during the current study available from the corresponding author on reasonable request.
