# Supplementary material for: Local niches explain coexistence in environmentally-distinct contact zones between Western Mediterranean vipers
Source: Sci Rep. 2023 Nov 30;13:21113. doi: 10.1038/s41598-023-48204-3 (PMC10689498; doi:10.1038/s41598-023-48204-3)
Supplement: Supplementary file 1 — Supplementary Information. [file 41598_2023_48204_MOESM1_ESM.docx]

**Supplementary Information**

**Title:** Local niches explain coexistence in environmentally-distinct contact zones between Western Mediterranean vipers

Inês Freitas* ^1,2,3^, Pedro Tarroso^1,2^, Óscar Zuazo^4^, Ricardo Zaldívar^5^, Javier Álvarez^6^, Manuel Meijide-Fuentes^7^, Federico Meijide^8^, Fernando Martínez-Freiría^1,2^

1. CIBIO, Centro de Investigação em Biodiversidade e Recursos Genéticos, InBIO Laboratório Associado, Campus de Vairão, Universidade do Porto, 4485-661 Vairão, Portugal;

2. BIOPOLIS Program in Genomics, Biodiversity and Land Planning, CIBIO, Campus de Vairão, 4485-661 Vairão, Portugal;

3. Departamento de Biologia, Faculdade de Ciências, Universidade do Porto, 4099-002 Porto, Portugal;

4. Calle La Puebla 1, 26250 Santo Domingo de la Calzada, Spain;

5. La Paz 89, 26004, Logroño, La Rioja, Spain;

6. Poniente 1, 26510, Pradejón, La Rioja, Spain;

7. Felicidad s/n, 42190, Golmayo, Soria;

8. Jueves La Saca s/n, 42003, Soria, Spain.

* corresponding author: [ifinesfreitas92@gmail.com](mailto:ifinesfreitas92@gmail.com)

***Details of eco-geographic variables (EGVs) used in ecological analyses***

**Supplementary Table S1**. List of eco-geographic variables (EGVs) used in ecological analyses. Code and name, units, range of variation (min – max) for climatic variables and average coverage percentage for landcover variables, for North Iberia and each contact zone are provided.

| **Code - name** | **units** | **North Iberia** | **High Ebro** | **Oja Tirón** | **Tierras Altas** |
| --- | --- | --- | --- | --- | --- |
| ISO - Isothermality | coef. | 316 - 376 | 365 - 375 | 365 - 372 | 351 - 365 |
| MinT - Min Temperature of Coldest Month | ºC | -6.8 - 5.7 | -1.6 - 1.4 | -6.4 - 2.1 | -4.6 - 1 |
| MeanT - Mean Temperature of Driest Quarter | ºC | 3.8 - 22.7 | 15.7 - 18.9 | 12.2 - 20.6 | 14.4 - 19.7 |
| APrec - Annual Precipitation | mm | 379 - 2188 | 598 - 1226 | 571 - 1014 | 433 - 912 |
| PrecS - Precipitation Seasonality | coef. | 13 - 41 | 23 - 38 | 21 - 35 | 18 - 29 |
| FOR - Forest | % | 29.4 | 28.8 | 29.0 | 25.4 |
| GRASS - Grasslands | % | 27.9 | 41.9 | 23.9 | 41.6 |
| CROP - Croplands | % | 25.4 | 7.2 | 34.4 | 11.6 |
| SHRUB - Shrublands | % | 13.5 | 20.0 | 10.2 | 18.8 |

***Transects parameters***

**Supplementary Table S2**. Transect parameters for each contact zone: total length of the transect in the coordinate system units (*L*), centre point (x,y) of the transect (*C*), angle of the transect in relation to a west-east line (in degrees) (*A*).

| **Contact zone** | **Transect length (*L*)** | **Transect center (*C*)** | **Transect angle (*A*)** |
| --- | --- | --- | --- |
| High Ebro | 12 | x = 0.5, y = -2.5 | 115 |
| Oja-Tirón | 12 | x = 0, y = 2.5 | 110 |
| Tierras Altas | 12 | x = 0, y = 0.4 | 100 |

***Ecological models’ performance***

**Supplementary Table S3**. Metrics of the 10 best model replicates, including average ± standard deviation TSS (on the top), Miller’ intercept (on the middle) and Miller’ slope (on the bottom) for training areas and projections, for both species (VAS – *V. aspis*, VLA – *V. latastei*) in each area (north Iberia – NIB, High Ebro – HE, Oja-Tirón – OT, Tierras Altas – TA). Acronyms for climatic and landcover variables follow Table S1.

|  |  | ***V. aspis*** | | | | ***V. latastei*** | | | |
| --- | --- | --- | --- | --- | --- | --- | --- | --- | --- |
| **Training area** | **Model type** | **NIB** | **HE** | **OT** | **TA** | **NIB** | **HE** | **OT** | **TA** |
| NIB | Climatic | .764 ± .013 | .157 ± .000 | .000 ± .000 | .476 ± .016 | .917 ± .025 | .773 ± .025 | .010 ± .014 | .000 ± .000 |
|  |  | .000 ± .000 | -.325 ± .129 | -1.141 ± .384 | -.157 ± .063 | -.108 ± .210 | -.627 ± .263 | .958 ± 1.033 | 3.113 ± 2.844 |
|  |  | 1.000 ± .000 | .198 ± .070 | .391 ± .129 | .915 ± .123 | .919 ± .259 | .634 ± .525 | -.314 ± .217 | -.466 ± .013 |
|  | Landcover | .080 ± .018 | -.169 ± .065 | .126 ± .039 | .116 ± .065 | .419 ± .017 | .243 ± .027 | .570 ± .011 | .292 ± .010 |
|  |  | .000 ± .000 | .456 ± .045 | .331 ± .099 | -.523 ± .101 | .000 ± .000 | -.096 ± .030 | -1.007 ± .067 | -.342 ± .013 |
|  |  | 1.000 ± .000 | -3.162 ± .529 | 1.905 ± .436 | 2.429 ± .572 | 1.000 ± .000 | .685 ± .084 | 1.804 ± .159 | .690 ± .039 |
|  | All | .777 ± .013 | .188 ± .017 | .000 ± .000 | .472 ± .010 | .940 ± .011 | .756 ± .098 | .071 ± .032 | .000 ± .000 |
|  |  | .000 ± .000 | -.534 ± .186 | -1.984 ± .241 | -.142 ± .052 | .431 ± .459 | -.191 ± .200 | -.193 ± .863 | .657 ± .903 |
|  |  | 1.000 ± .000 | .289 ± .082 | .733 ± .054 | .849 ± .111 | .066 ± .221 | .393 ± .462 | -.040 ± .089 | -.123 ± .008 |
| HE | Climatic | .123 ± .006 | .712 ± .043 | -.051 ± .073 | .000 ± .000 | .828 ± .023 | .823 ± .032 | -.006 ± .014 | .000 ± .000 |
|  |  | 1.286 ± .041 | .000 ± .000 | -9.094 ± 5.401 | 19.377 ± 38.151 | -.247 ± 1.012 | .000 ± .000 | 6.390 ± 3.199 | -1.190 ± 3.123 |
|  |  | .058 ± .004 | 1.000 ± .000 | -1.337 ± .372 | .673 ± 1.171 | .349 ± .077 | 1.000 ± .000 | -.748 ± .262 | -.001 ± .267 |
|  | Landcover | -.008 ± .014 | .395 ± .055 | .200 ± .018 | -.080 ± .028 | .290 ± .065 | .426 ± .039 | .393 ± .186 | .188 ± .085 |
|  |  | .018 ± .010 | .000 ± .000 | 1.046 ± .149 | -.009 ± .087 | .194 ± .058 | .000 ± .000 | .382 ± .478 | .065 ± .017 |
|  |  | .023 ± .010 | 1.000 ± .000 | .482 ± .123 | -.017 ± .118 | .328 ± .046 | 1.000 ± .000 | .988 ± .229 | .259 ± .087 |
|  | All | .110 ± .005 | .686 ± .042 | -.006 ± .009 | .000 ± .000 | .764 ± .122 | .820 ± .018 | .023 ± .051 | -.06 ± .134 |
|  |  | 1.181 ± .057 | .000 ± .000 | -9.904 ± 4.653 | .000 ± .000 | -.366 ± 1.174 | .000 ± .000 | -1.940 ± 3.762 | -4.050 ± 4.172 |
|  |  | .051 ± .004 | 1.000 ± .000 | -1.147 ± .283 | NA | .302 ± .052 | 1.000 ± .000 | .176 ± .354 | .227 ± .393 |
| OT | Climatic | .024 ± .013 | .000 ± .000 | .915 ± .014 | .000 ± .000 | -.039 ± .010 | .000 ± .531 | .941 ± .012 | NA |
|  |  | .139 ± .033 | -8.866 ± 6.067 | .000 ± .000 | .000 ± .000 | -.014 ± .083 | .0568 ± .014 | .000 ± .000 | .000 ± .000 |
|  |  | -.009 ± .001 | -2.250 ± .168 | 1.000 ± .000 | NA | -.014 ± .083 | .056 ± .014 | 1.000 ± .000 | .000 ± .000 |
|  | Landcover | .084 ± .007 | .052 ± .022 | .489 ± .026 | .236 ± .029 | .337 ± .089 | .226 ± .153 | .676 ± .032 | .264 ± .085 |
|  |  | .048 ± .011 | -.428 ± .090 | .000 ± .000 | .003 ± .012 | .315 ± .150 | .618 ± .097 | .000 ± .000 | .521 ± .127 |
|  |  | -.014 ± .002 | .138 ± .026 | 1.000 ± .000 | -.001 ± .007 | .315 ± .150 | .618 ± .097 | 1.000 ± .000 | .521 ± .127 |
|  | All | .038 ± .022 | .000 ± .000 | .929 ± .014 | .000 ± .000 | -.054 ± .014 | .000 ± .947 | .954 ± .014 | NA |
|  |  | .100 ± .057 | -55.609 ± 42.502 | .000 ± .000 | .000 ± .000 | .056 ± .208 | .075 ± .027 | .023 ± .140 | .000 ± .000 |
|  |  | -.006 ± .003 | -1.550 ± 1.177 | 1.000 ± .000 | NA | .056 ± .208 | .075 ± .027 | 1.000 ± .000 | .000 ± .000 |
| TA | Climatic | .538 ± .027 | .000 ± .000 | .000 ± .000 | .778 ± .038 | .373 ± .060 | .000 ± .000 | .000 ± .000 | .906 ± .046 |
|  |  | -.367 ± .062 | .019 ± .000 | 7.426 ± 2.951 | .000 ± .000 | .438 ± .300 | .000 ± .000 | -53.340 ± 49.003 | .001 ± .006 |
|  |  | .039 ± .005 | NA | -.428 ± .269 | 1.000 ± .000 | .029 ± .011 | NA | -2.469 ± .218 | 1.048 ± .098 |
|  | Landcover | .070 ± .015 | -.242 ± .036 | .163 ± .052 | .373 ± .056 | .319 ± .032 | .099 ± .139 | .705 ± .010 | .345 ± .068 |
|  |  | .095 ± .035 | -.294 ± .095 | .869 ± .059 | .000 ± .000 | -.165 ± .047 | .108 ± .044 | -1.296 ± .078 | .000 ± .000 |
|  |  | .141 ± .048 | -.686 ± .218 | .562 ± .069 | 1.000 ± .000 | .403 ± .058 | .336 ± .156 | .713 ± .090 | 1.000 ± .000 |
|  | All | .523 ± .023 | .000 ± .000 | .000 ± .000 | .823 ± .046 | .366 ± .125 | .000 ± .000 | .002 ± .004 | .941 ± .036 |
|  |  | -.389 ± .154 | .019 ± .000 | 9.960 ± 12.230 | .000 ± .000 | .671 ± .658 | .000 ± .000 | 12.268 ± 27.433 | -.002 ± .011 |
|  |  | .035 ± .005 | NA | -.384 ± .297 | 1.000 ± .000 | .035 ± .023 | NA | .566 ± .984 | 1.047 ± .078 |

***Maps of probability of occurrence for V. aspis and V. latastei in North Iberia and the three contact zones***


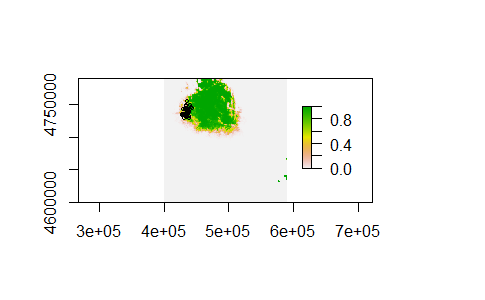

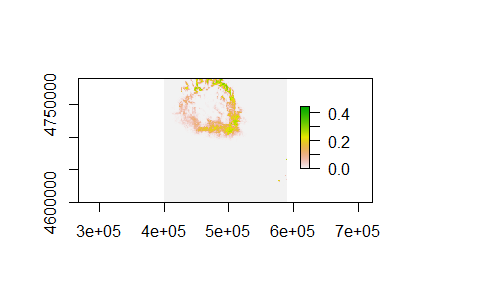

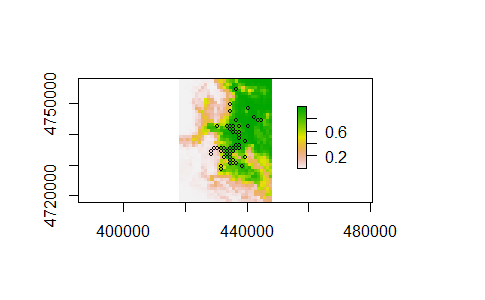

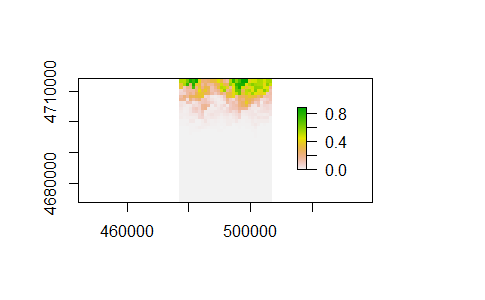

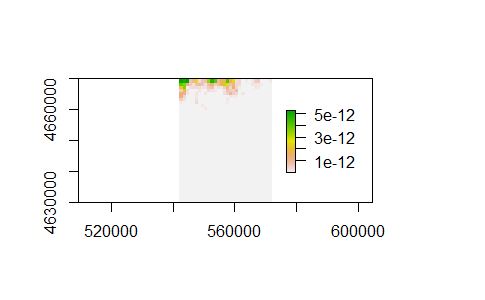

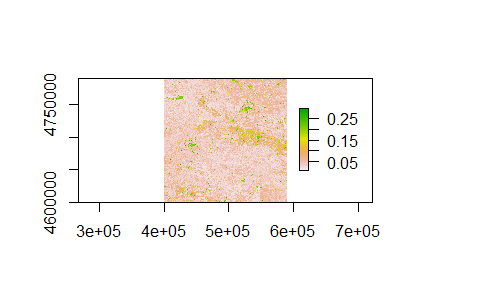

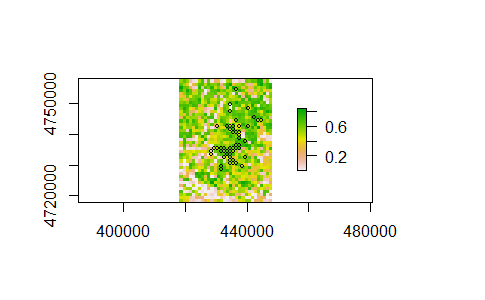

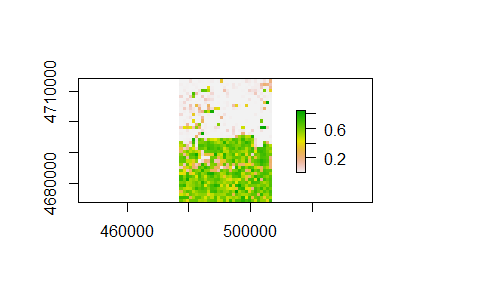

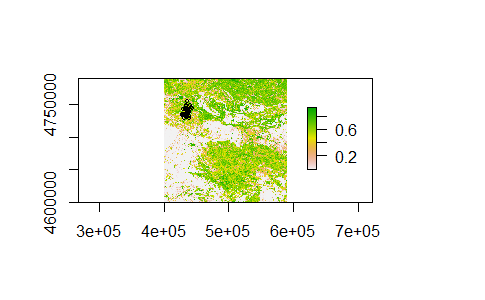

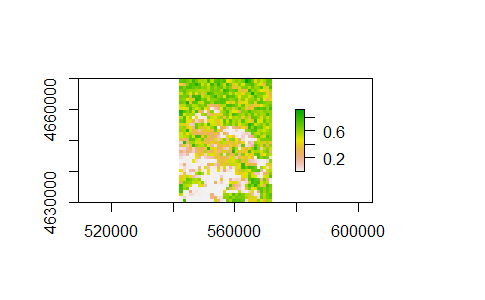

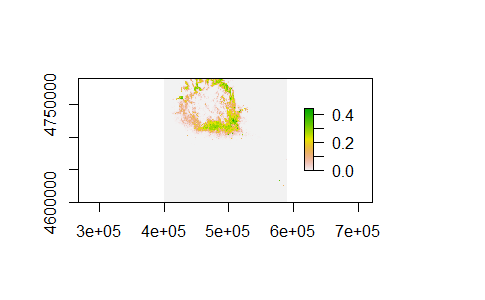

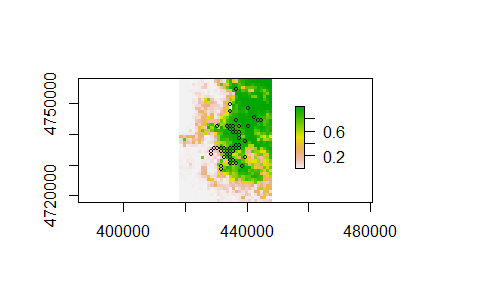

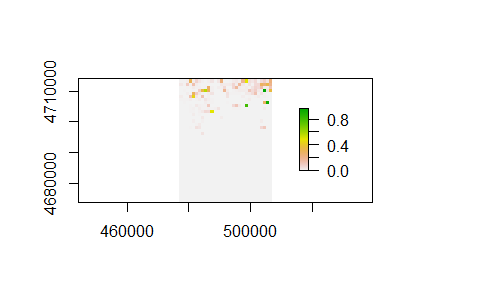

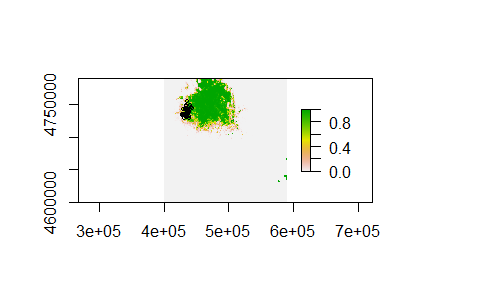

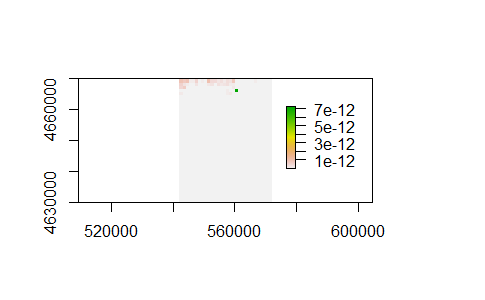


*V. aspis:* Training area High Ebro

High Ebro

Projection to North Iberia

Projection to Oja Tirón

Projection to Tierras Altas

Standard deviation

High Ebro

Projection to North Iberia

Projection to Oja Tirón

Projection to Tierras Altas

Standard deviation

High Ebro

Projection to North Iberia

Projection to Oja Tirón

Projection to Tierras Altas

Standard deviation

A

B

C

**
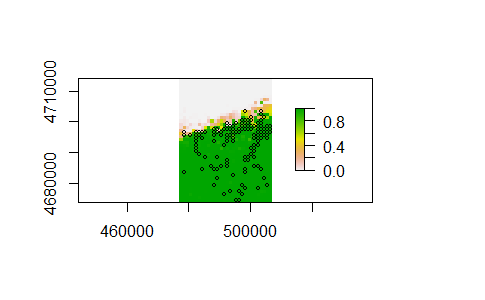

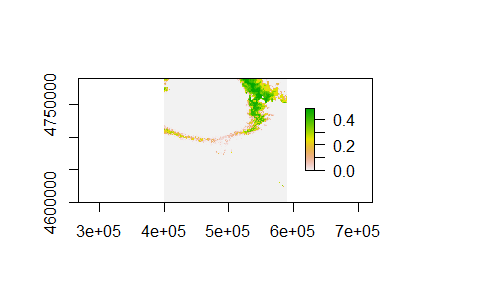

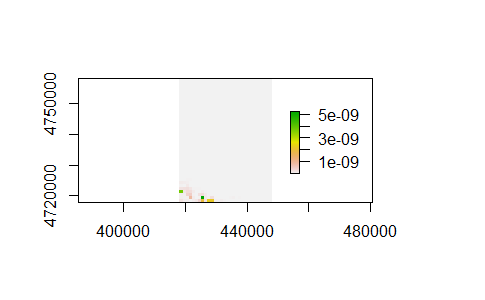

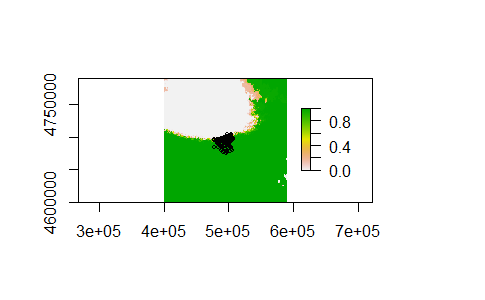

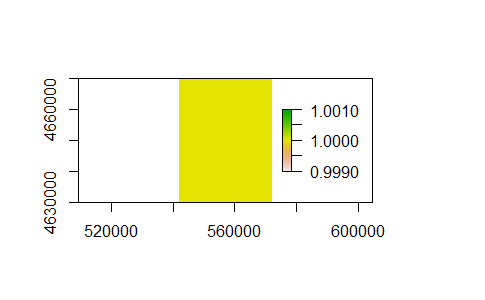

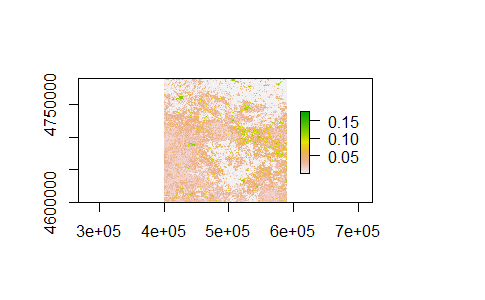

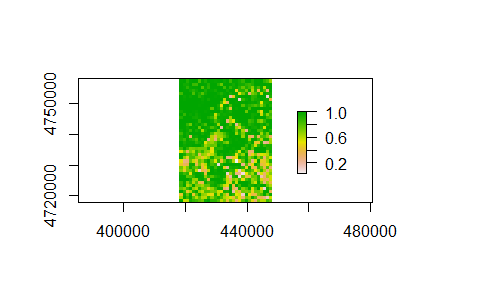

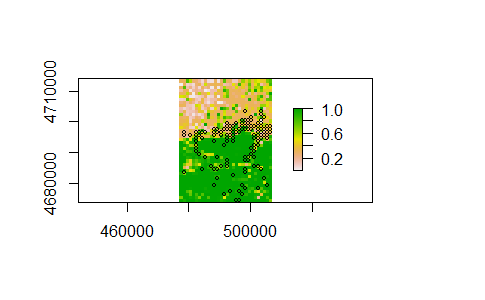

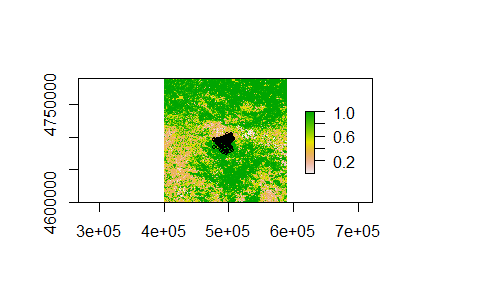

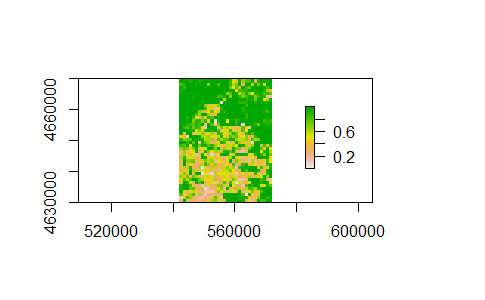

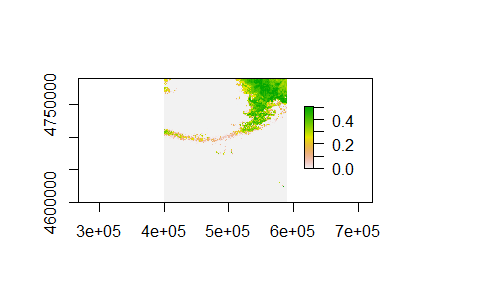

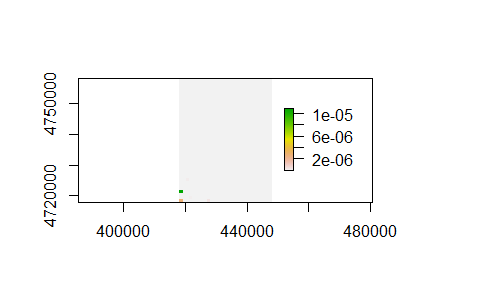

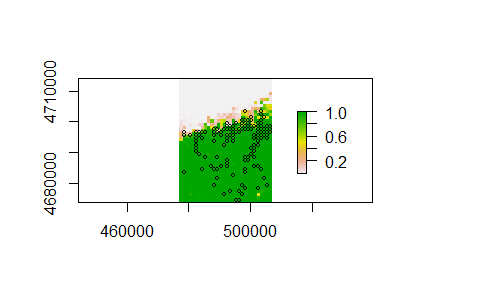

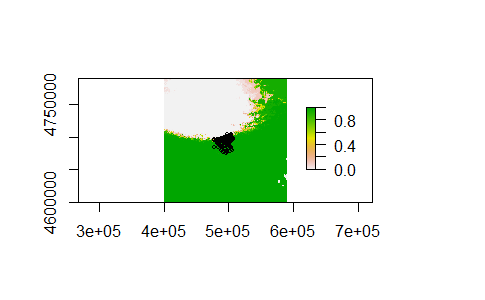

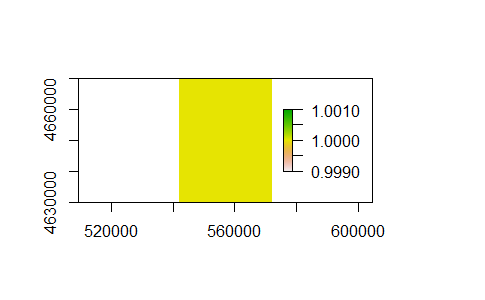
**

*V. aspis:* Training area Oja-Tirón

Projection to North Iberia

Projection to Tierras Altas

Standard deviation

Projection to North Iberia

Projection to High Ebro

Projection to Tierras Altas

Standard deviation

Projection to North Iberia

Projection to High Ebro

Projection to Tierras Altas

Standard deviation

A

C

Oja Tirón

Projection to High Ebro

B

Oja Tirón

Oja Tirón

**
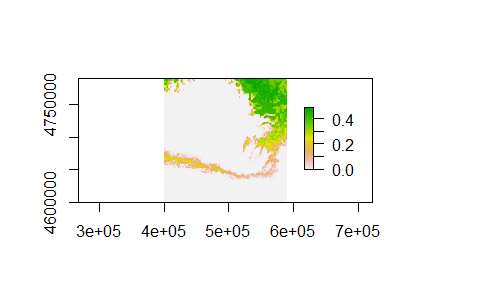

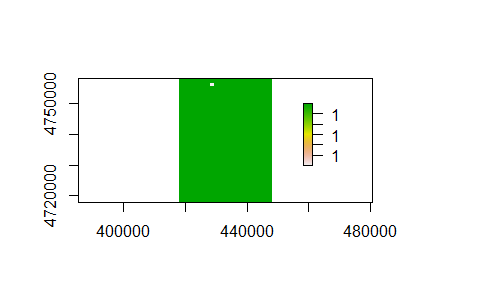

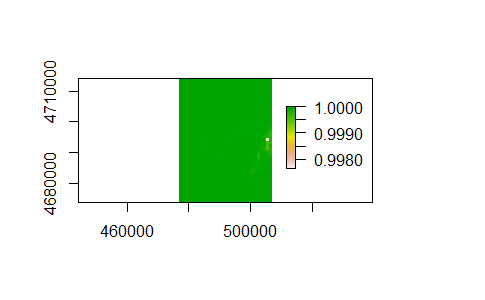

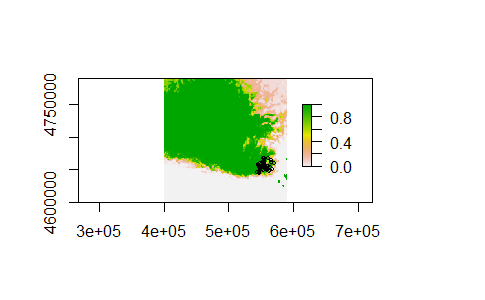

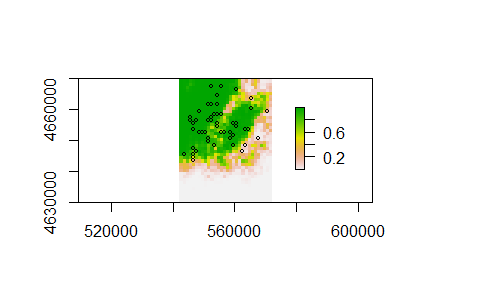

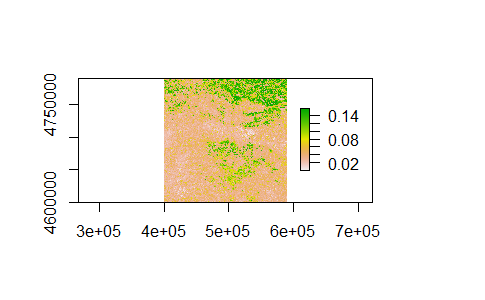

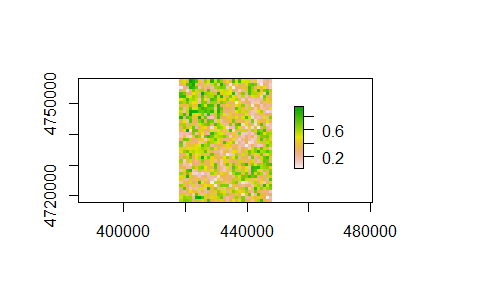

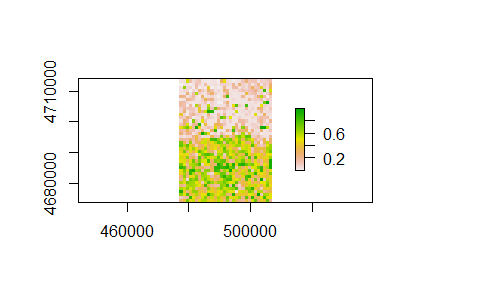

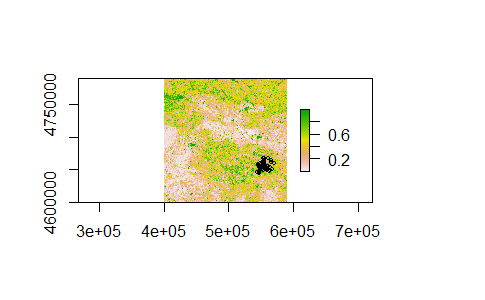

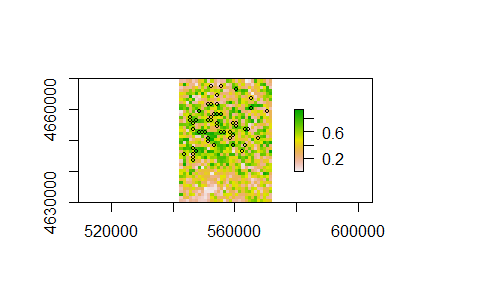

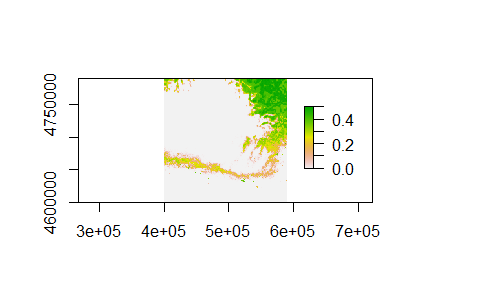

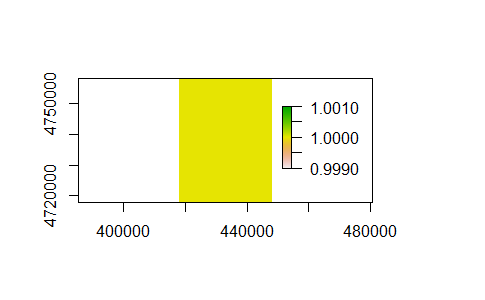

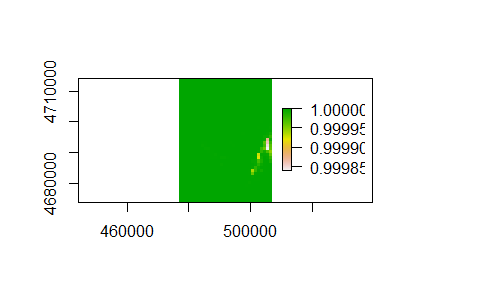

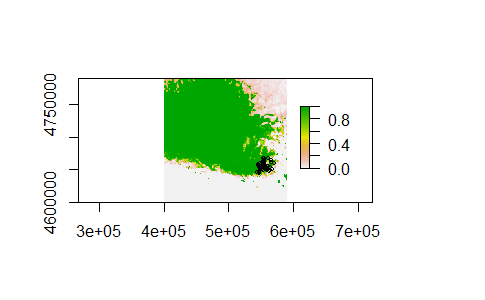

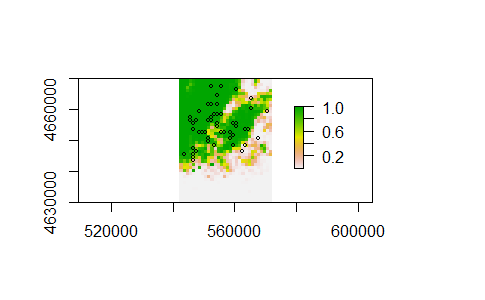
**

*V. aspis:* Training area Tierras Altas

Projection to North Iberia

Standard deviation

Projection to North Iberia

Projection to High Ebro

A

C

Tierras Altas

Projection to High Ebro

B

Tierras Altas

Tierras Altas

Projection to Oja Tirón

Projection to North Iberia

Projection to High Ebro

Projection to Oja Tirón

Standard deviation

Standard deviation

Projection to Oja Tirón

**
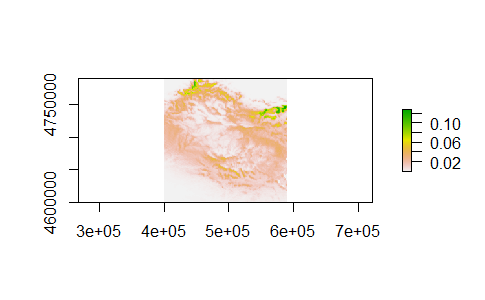

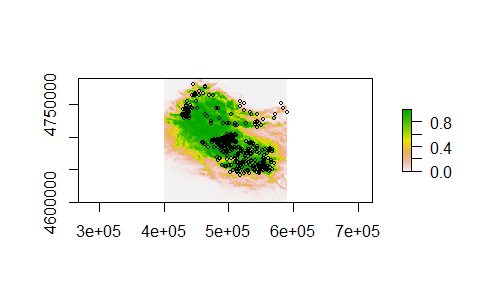

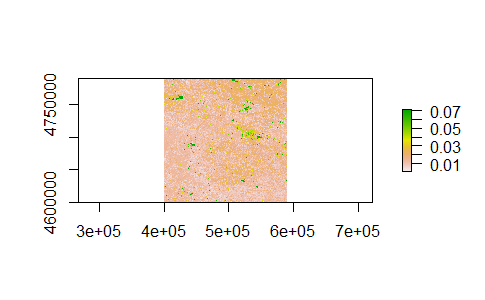

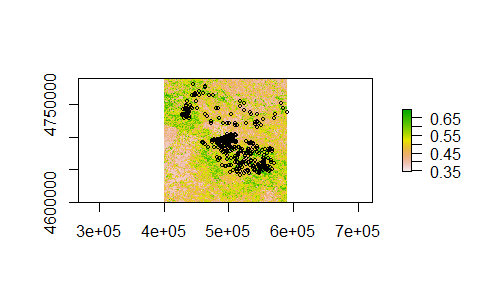

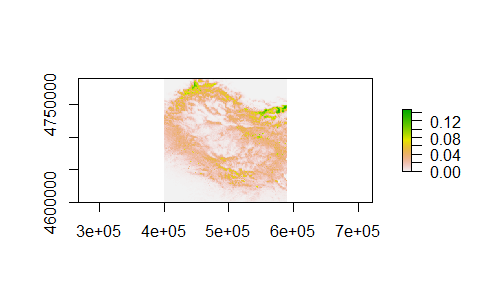

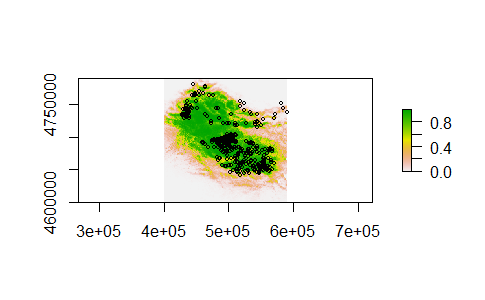
**

*V. aspis:* Training area North Iberia

North Iberia

A

C

B

Standard deviation

North Iberia

Standard deviation

North Iberia

Standard deviation

**
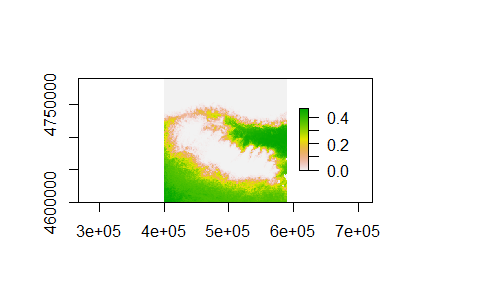

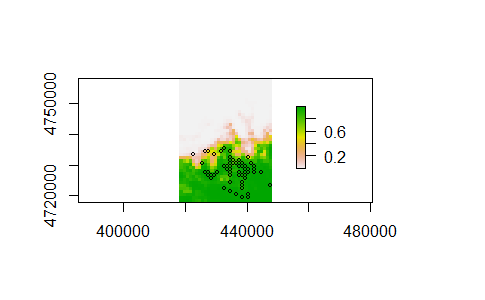

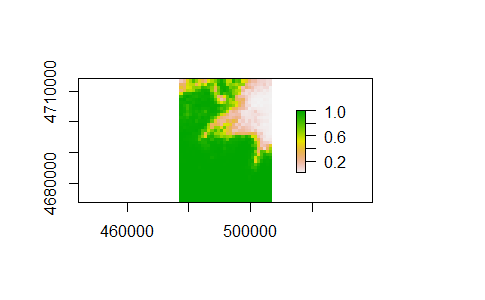

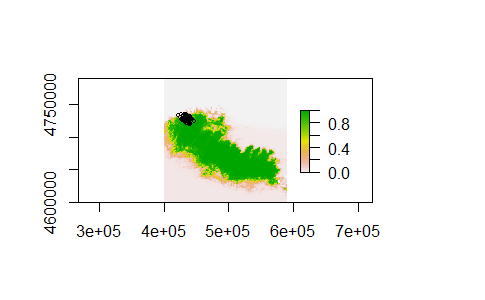

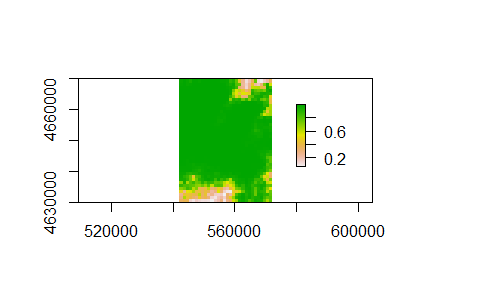

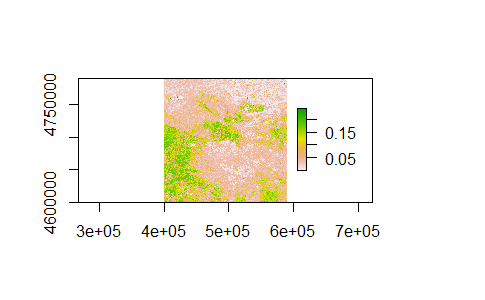

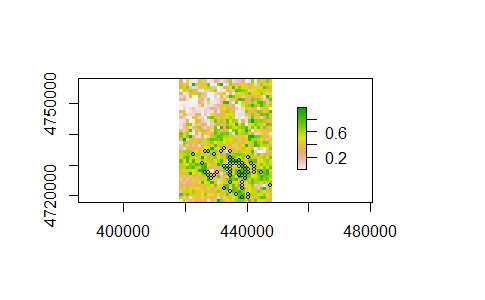

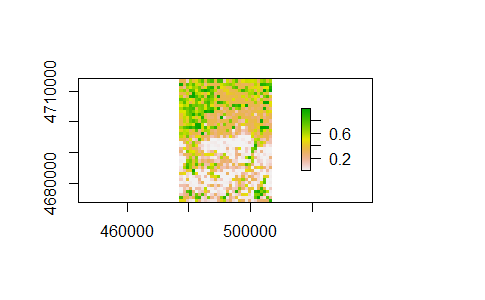

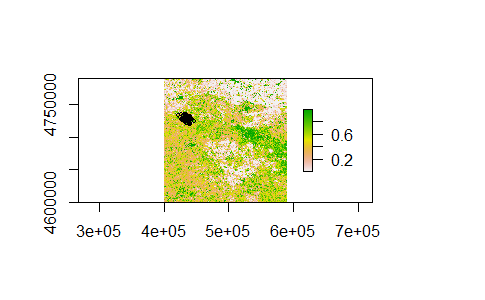

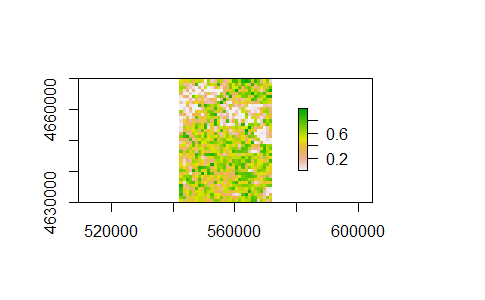

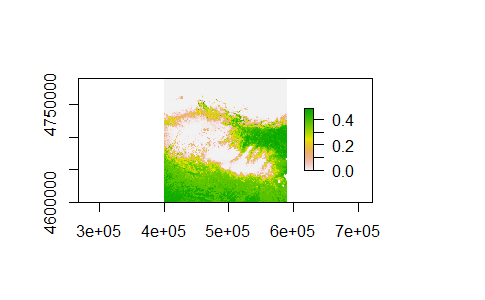

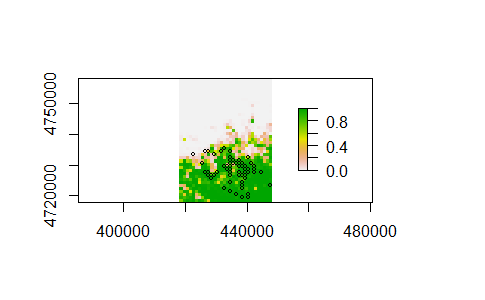

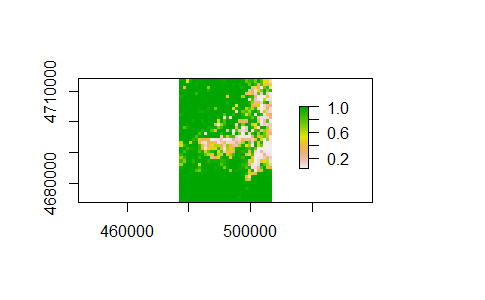

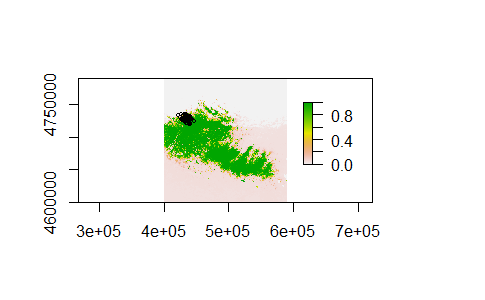

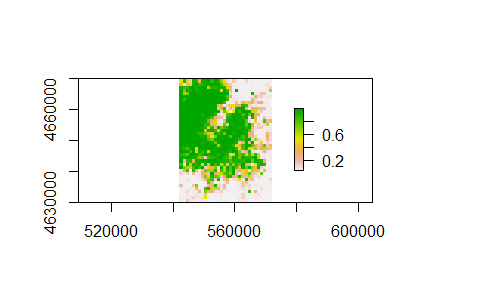
**

*V. latastei:* Training area High Ebro

High Ebro

Projection to North Iberia

Projection to Tierras Altas

Standard deviation

Projection to North Iberia

Standard deviation

High Ebro

Projection to North Iberia

Projection to Oja Tirón

Projection to Tierras Altas

Standard deviation

A

B

C

Projection to Oja Tirón

High Ebro

Projection to Oja Tirón

Projection to Tierras Altas

**
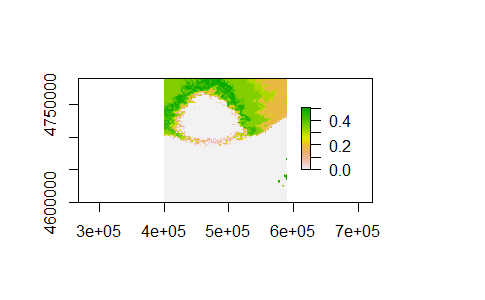

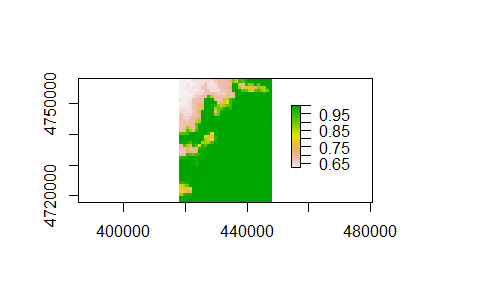

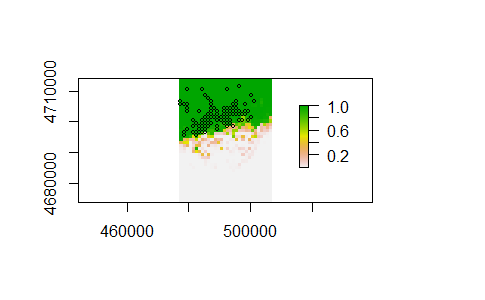

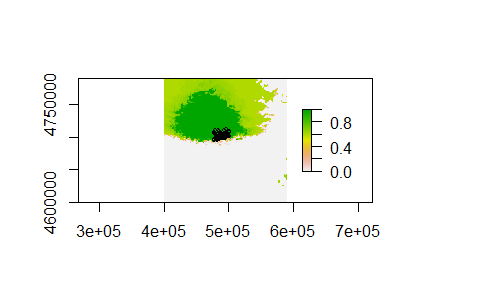

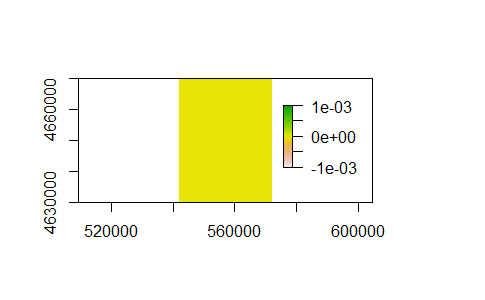

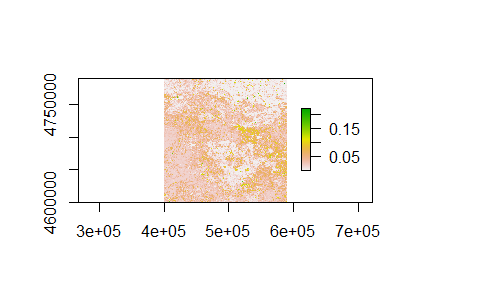

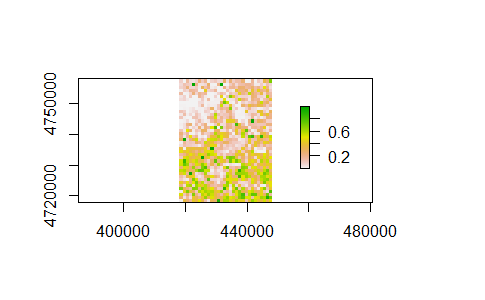

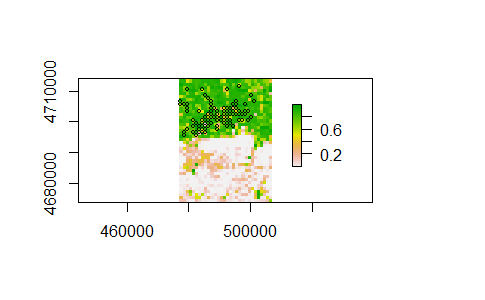

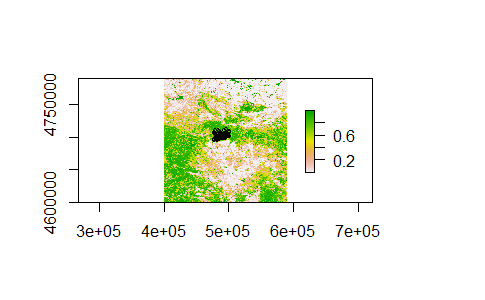

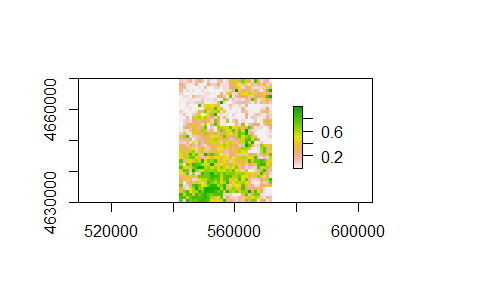

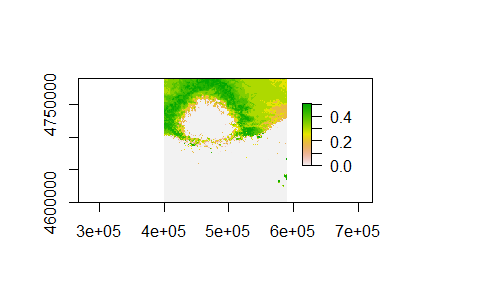

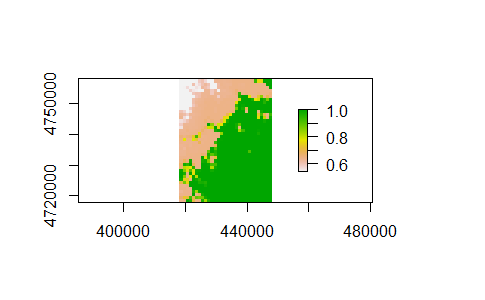

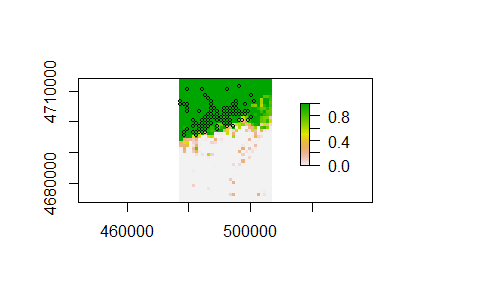

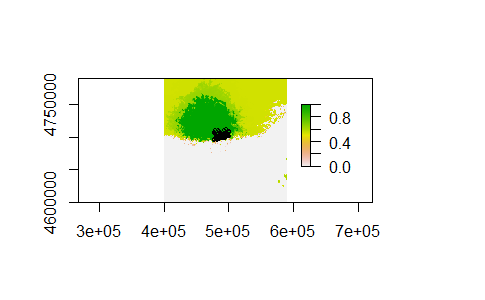

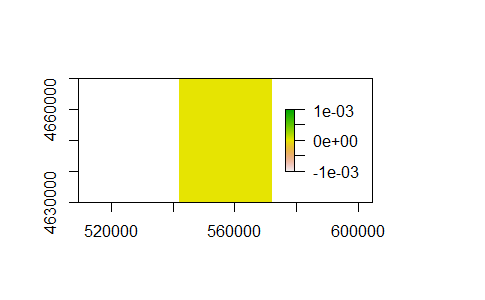
**

*V. latastei:* Training area Oja Tirón

Standard deviation

Projection to North Iberia

Projection to Tierras Altas

Standard deviation

Projection to North Iberia

Projection to High Ebro

Standard deviation

A

Oja Tirón

Oja Tirón

Projection to North Iberia

B

Projection to High Ebro

Projection to Tierras Altas

Oja Tirón

C

Projection to High Ebro

Projection to Tierras Altas

**
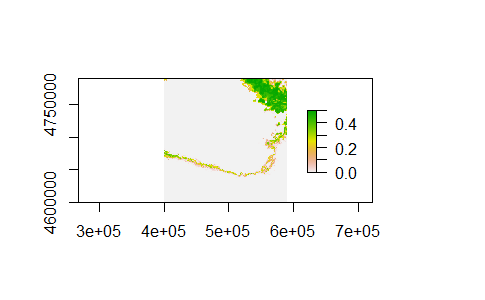

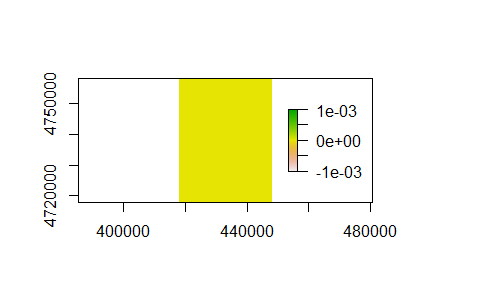

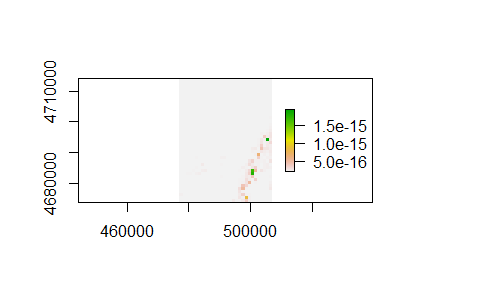

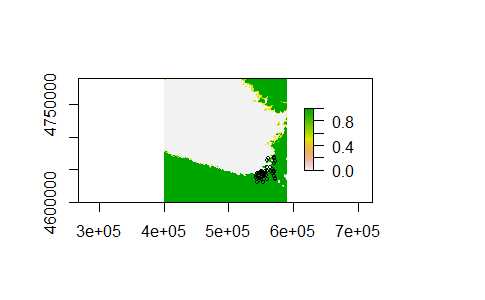

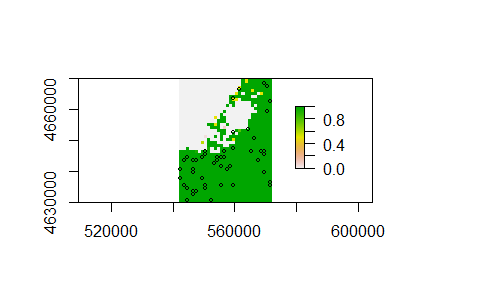

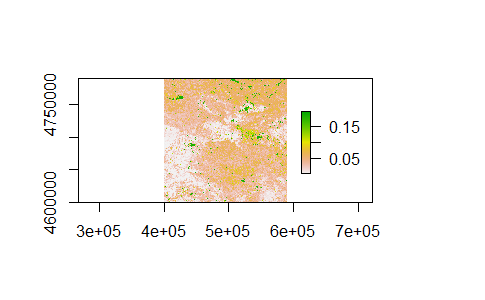

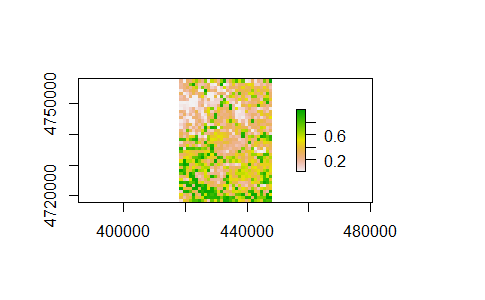

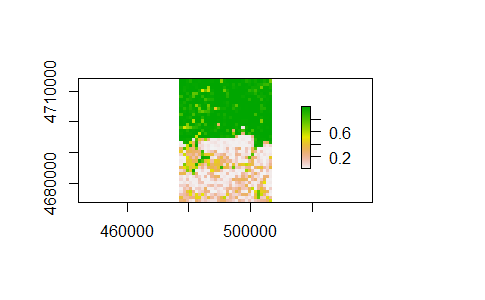

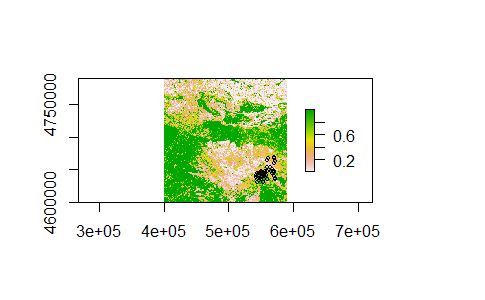

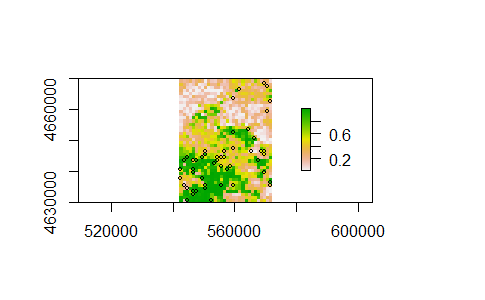

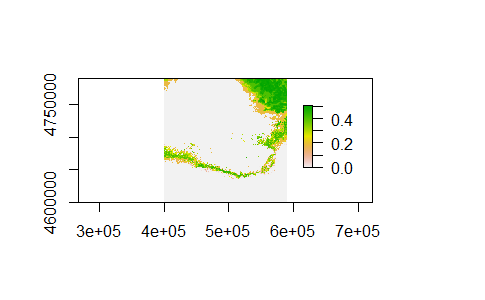

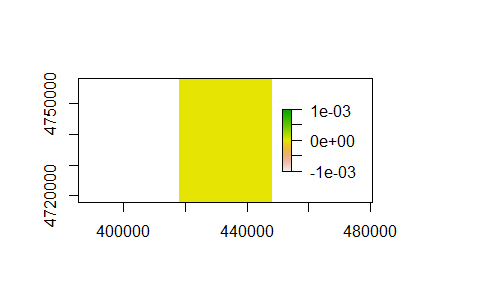

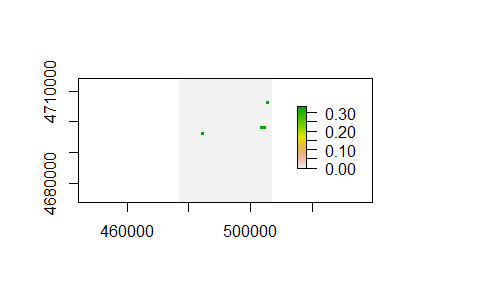

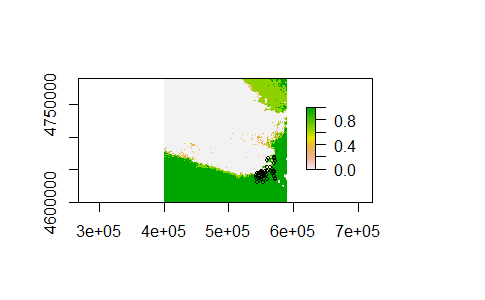

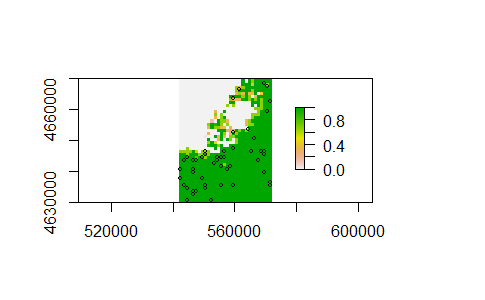
**

*V. latastei:* Training area Tierras Altas

Standard deviation

Projection to North Iberia

Projection to High Ebro

A

C

Tierras Altas

Tierras Altas

Projection to High Ebro

Standard deviation

Standard deviation

Projection to North Iberia

B

Projection to High Ebro

Tierras Altas

Projection to Oja Tirón

Projection to North Iberia

Projection to Oja Tirón

Projection to Oja Tirón

**
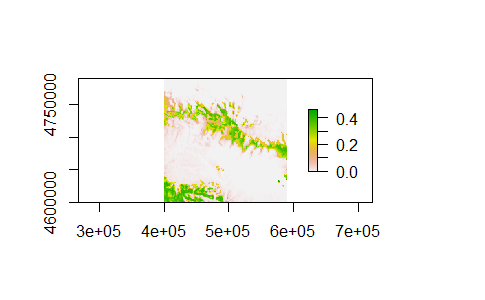

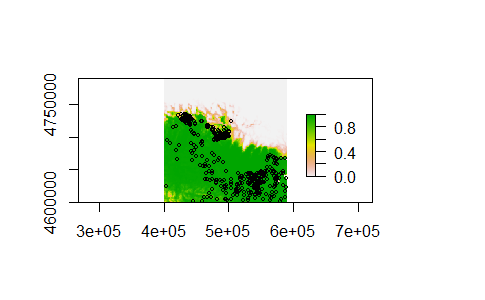

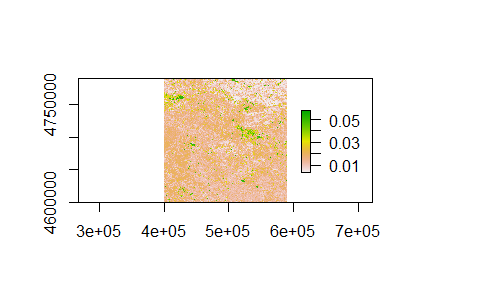

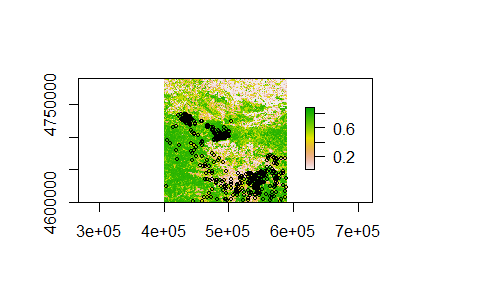
**

*V. latastei:* Training area North Iberia

North Iberia

A

C

B

Standard deviation

North Iberia

Standard deviation

North Iberia

Standard deviation

**Supplementary Figure S1**. Models of probability of occurrence of *V. aspis* and *V. latastei* in North Iberia and the three contact zones (High Ebro, Oja-Tirón and Tierras Altas), using climatic (A), landcover (B) and climatic + landcover (C) variables. Model projections to the other areas and standard deviation maps are presented. The occurrences datasets used to model the species distributions are depicted.

**Supplementary Text S1.** Description of the four training areas, North Iberia, the High Ebro, Oja-Tirón and Tierras Altas. Detailed maps of the species’ spatial distribution in each contact zone are provided. Occurrence data of *V. aspis* is depicted in blue, *V. latastei* in red, *V. seoanei* in grey and sympatry in yellow. Google Satellite was used as a background layer. Three rasters representing the climatic and landcover variation in the four areas are also provided. Bioclimatic variables, mean annual air temperature (Bio 1) and annual precipitation amount (Bio 12), were downloaded from CHELSA v1.2 (<https://chelsa-climate.org/>) and a landcover raster, representing the percentual cover of the main land types was obtained from Copernicus (<https://www.copernicus.eu/>).

North Iberia

North Iberia is located in north-central Spain with a total area of 36,100 km^2^, where *V. aspis*, *V. latastei* and *V. seoanei* establish contact broadly (see Fig. 1). *V. seoanei*, is a viper species with Euro-Siberian affinity and phylogenetically-distant from the western Mediterranean vipers, *V. aspis* and *V. latastei* [1]. It is found in the northern region of this area (and in the High Ebro contact zone, see below), restricted to areas with marked Atlantic climate [2,3]. Climate is characterized by low to mild annual temperatures (mean annual temperature range from 3.8 to 22.7 ºC) and low to high levels of precipitation (annual rainfall of 379 to 2188 mm/year), with altitude ranging from 7 to 2147 m. The most prevalent landcover types are forests (30%), grasslands (28%) and croplands (25%) [4,5].

**Annual precipitation**

**Mean annual temperature**

**Precipitation**

**Temperature**

**Landcover**

**Closed forest**

**Open forest**

**Shrubland**

**Herbaceous vegetation**

**Herbaceous wetland**

**Moss and lichen**

**Bare/sparse vegetation**

**Cropland**

**Built-up**

High Ebro

The High Ebro is a contact zone between the three Iberian vipers, where the two Western Mediterranean vipers, *V. aspis* and *V. latastei*, meet in sympatry, while *V. seoanei* is allopatrically distributed [6].

This contact zone is located in the upper course of the Ebro river, consists of plateaus and steep valleys formed by Ebro river and its tributaries (altitude ranging from 590 to 1256 m), and it is embedded in a vast natural landscape with low human disturbance. Climate is characterised by low annual temperature (-1.6 - 18.9 ºC) and moderate levels of precipitation (598 - 1226 mm/year) [6,7,8,9]. The landscape is mostly characterized by vast grasslands (42%), forested areas (30%) and shrublands (20%) [4,5].

**Landcover**

**Annual precipitation**

**Mean annual temperature**

**Precipitation**

**Temperature**

Oja-Tirón

Climate is characterized by low annual temperature (4.1 - 12.5 ºC) and moderate levels of precipitation (571-1015 mm/year), with altitude ranging from 480 to 2271 m (Zuazo et al. 2019).

**Closed forest**

**Open forest**

**Shrubland**

**Herbaceous vegetation**

**Herbaceous wetland**

**Moss and lichen**

**Bare/sparse vegetation**

**Cropland**

**Built-up**

Oja-Tirón

The Oja-Tirón contact zone, located in the north-western slopes of the Iberian System, is shaped by Tirón and Oja rivers (altitude ranging from 480 to 2271 m), and consists mostly of intensive culture fields of cereals [10]. Climate is characterized by low annual temperature (4.1 - 12.5 ºC) and moderate levels of precipitation (604-1016 mm/year). In the northern half of this area, the landscape is mostly covered by croplands (42%), while in the southern half it is covered by a similar proportion of forests (30%) and grasslands (24%) [4,5].

**Annual precipitation**

**Mean annual temperature**

**Landcover**

**Precipitation**

**Temperature**

**Closed forest**

**Open forest**

**Shrubland**

**Herbaceous vegetation**

**Herbaceous wetland**

**Moss and lichen**

**Bare/sparse vegetation**

**Cropland**

**Built-up**

Tierras Altas

The Tierras-Altas contact zone, located in the south-eastern slopes of the Iberian System, is shaped by Tera river running southwards and Cidacos and Linares rivers running north-eastwards (altitude ranging from 724 to 1720); its landscape is mostly dominated by abandoned grazing fields. Climate is characterized by low annual temperature (-4.6 - 19.7 ºC) and low levels of precipitation (433 - 912 mm/year). The most prevalent landcover type is grasslands (42%), followed by forest (26%) and shrublands (19%) [4,5].

**Landcover**

**Annual precipitation**

**Mean annual temperature**

**Precipitation**

**Temperature**

**Closed forest**

**Open forest**

**Shrubland**

**Herbaceous vegetation**

**Herbaceous wetland**

**Moss and lichen**

**Bare/sparse vegetation**

**Cropland**

**Built-up**

**References**

[1] Freitas, I. *et al.* Evaluating taxonomic inflation: towards evidence-based species delimitation in Eurasian vipers (Serpentes: Viperinae). *Amphib-reptil.* **41**, 1–27 (2020).

[2] Martínez‐Freiría, F., Velo‐Antón, G. & Brito, J. C. Trapped by climate: interglacial refuge and recent population expansion in the endemic Iberian adder *Vipera seoanei*. *Divers. Distrib.* **21**, 331-344 (2015).

[3] Chamorro, D., Martínez‐Freiría, F., Real, R. & Muñoz, A. R. Understanding parapatry: How do environment and competitive interactions shape Iberian vipers’ distributions?. *J. Biogeogr.* **48**, 1322-1335 (2021).

[4] Karger, D.N. *et al*. Climatologies at high resolution for the Earth land surface areas. *Sci. Data*. **4**, 170122 (2017).

[5] Buchhorn, M. *et al*. Copernicus global land cover layers—collection 2. *Remote Sens.* **12**, 1044 (2020).

[6] Martínez-Freiría, F., Sillero, N., Lizana, M. & Brito, J.C. GIS-based niche models identify environmental correlates sustaining a contact zone between European vipers. *Divers. Distrib.* **14**, 452-461 (2008).

[7] Martínez-Freiría, F., Brito, J. C. & Avia, M. L. Intermediate forms and syntopy among vipers (*Vipera aspis* and *V. latastei*) in Northern Iberian Peninsula. *Herpetol. Bull*. **97**, 14 (2006).

[8] Martínez‐Freiría, F., Santos, X., Pleguezuelos, J. M., Lizana, M. & Brito, J. C. Geographical patterns of morphological variation and environmental correlates in contact zones: A multi‐scale approach using two Mediterranean vipers (Serpentes). *J. Zool. Syst. Evol. Res.* **47**, 357-367 (2009).

[9] Martínez-Freiría, F., Lizana, M., do Amaral, J. P. & Brito, J. C. Spatial and temporal segregation allows coexistence in a hybrid zone among two Mediterranean vipers (*Vipera aspis* and *V. latastei*). *Amphib-reptil.* **31**, 195-212 (2010).

[10] Zuazo, Ó., Freitas, I., Zaldívar, R. & Martínez-Freiría, F. Coexistence and intermediate morphological forms between *Vipera aspis* and *V. latastei* in the intensive agriculture fields of north-western Iberian System. *Bol. Asoc. Herpetol. Esp*. **30**, 35-41 (2019).

**Supplementary Text S2. Details on species occurrences collection and environmental factors selection.**

**Occurrence records**

In the three contact zones, species occurrences were obtained along spatially and environmentally stratified sampling campaigns, developed along different but overlapping time spans: 1) in the High Ebro, occurrences were obtained between 2004 and 2020, and include both published [1,2,3,4] and unpublished data (posteriorly to 2008); 2) in Oja-Tirón, occurrences were obtained from 2015 to 2020, and include mostly published data (up to 2019, 82 % of the data) [5]; and 3) in Tierras-Altas, occurrences were obtained from 2000 to 2017, and include unpublished data. Species occurrences along the remaining part of North Iberia were obtained through opportunistic fieldwork performed by the authors and collaborators (n=301; see Acknowledgements), specimens stored in three museum collections (n=42; see Acknowledgements) and records provided by the Spanish Herpetological Association (AHE) (n=88). Time span for these records ranges from 1981 to 2020.

**Environmental factors**

A total of 23 continuous eco-geographical variables (EGVs), including 19 bioclimatic (temperature and precipitation related) and four landcover variables were initially considered. Bioclimatic variables were downloaded from CHELSA v1.2 (https://chelsa-climate.org/), with a spatial resolution of 1x1 km [6]. Land cover variables, representing the main vegetation categories of the three contact zones, were obtained from Copernicus (https://www.copernicus.eu/) and consisted of a set of four continuous layers depicting the percentual cover of the main land cover types in a pixel, at 100x100 m resolution for the referenced year of 2015 [7]. The resolution of the land cover variables was upscaled to 1x1 km, using R aggregate function to obtain the average value of the input cells. A set of nine bioclimatic and four landcover variables were selected (Table S1) after testing for correlation (*R <* 0*.*68). All variables are biological relevant for the species and were previously deemed important for the ecological modelling of these vipers at distinct scales [2,8,9,10].

**References**

[1] Martínez-Freiría, F., Brito, J. C. & Avia, M. L. Intermediate forms and syntopy among vipers (*Vipera aspis* and *V. latastei*) in Northern Iberian Peninsula. *Herpetol. Bull*. **97**, 14 (2006).

[2] Martínez-Freiría, F., Sillero, N., Lizana, M. & Brito, J.C. GIS-based niche models identify environmental correlates sustaining a contact zone between European vipers. *Divers. Distrib.* **14**, 452-461 (2008).

[3] Martínez-Freiría, F., Lizana, M., do Amaral, J. P. & Brito, J. C. Spatial and temporal segregation allows coexistence in a hybrid zone among two Mediterranean vipers (*Vipera aspis* and *V. latastei*). *Amphib-reptil.* **31**, 195-212 (2010).

[4] Tarroso, P., Pereira, R. J., Martínez‐Freiría, F., Godinho, R. & Brito, J. C. Hybridization at an ecotone: ecological and genetic barriers between three Iberian vipers. *Mol. Ecol.* **23**, 1108-1123 (2014).

[5] Zuazo, Ó., Freitas, I., Zaldívar, R. & Martínez-Freiría, F. Coexistence and intermediate morphological forms between *Vipera aspis* and *V. latastei* in the intensive agriculture fields of north-western Iberian System. *Bol. Asoc. Herpetol. Esp*. **30**, 35-41 (2019).

[6] Karger, D.N. *et al*. Climatologies at high resolution for the Earth land surface areas. *Sci. Data*. **4**, 170122 (2017).

[7] Buchhorn, M. *et al*. Copernicus global land cover layers—collection 2. *Remote Sens.* **12**, 1044 (2020).

[8] Martínez‐Freiría, F. *et al.* Climatic refugia boosted allopatric diversification in western Mediterranean vipers. *J. Biogeogr.* **47**, 1698-1713 (2020).

[9] Chamorro, D., Martínez‐Freiría, F., Real, R. & Muñoz, A. R. Understanding parapatry: How do environment and competitive interactions shape Iberian vipers’ distributions?. *J. Biogeogr.* **48**, 1322-1335 (2021).

[10] Scaramuzzi, A., Freitas, I., Sillero, N. & Martínez‐Freiría, F. Meso‐habitat distribution patterns and ecological requirements of two Mediterranean vipers depict weak competition in a contact zone. *J. Zool.* (2023).

**Supplementary Text S3.** Coefficients’ estimate, standard error (Std. Error), z value and p-value (Pr(>|z|) for the predictor variables in the models. Significance codes are as follows: 0 (***), 0.001 (**), 0.01 (*), 0.05 (.), below 0.05 ( ).

1. Models developed for *V. aspis* in the High Ebro (with climatic, landcover and both variable types)

Climatic

|  | **Estimate** | **Std. Error** | **Adjusted SE** | **z value** | **Pr(>\|z\|)** |  |
| --- | --- | --- | --- | --- | --- | --- |
| **(Intercept)** | 0.000 | 0.000 | 0.000 | NA | NA |  |
| **ISO** | 4.959 | 1.240 | 1.258 | 3.941 | 0.000 | *** |
| **MinT** | 8.134 | 2.894 | 2.936 | 2.770 | 0.006 | ** |
| **MeanT** | -4.952 | 3.567 | 3.620 | 1.368 | 0.171 |  |
| **APrec** | -1.458 | 2.098 | 2.128 | 0.685 | 0.493 |  |
| **PrecS** | -1.409 | 1.012 | 1.026 | 1.374 | 0.169 |  |

Landcover

|  | **Estimate** | **Std. Error** | **Adjusted SE** | **z value** | **Pr(>\|z\|)** |
| --- | --- | --- | --- | --- | --- |
| **(Intercept)** | 0.000 | 0.000 | 0.000 | NA | NA |
| **FOR** | -0.668 | 3.277 | 3.321 | 0.201 | 0.841 |
| **CROP** | -2.273 | 2.324 | 2.358 | 0.964 | 0.335 |
| **SHRUB** | 0.675 | 1.502 | 1.521 | 0.444 | 0.657 |
| **GRASS** | -2.047 | 2.405 | 2.437 | 0.840 | 0.401 |

All

|  | **Estimate** | **Std. Error** | **Adjusted SE** | **z value** | **Pr(>\|z\|)** |  |
| --- | --- | --- | --- | --- | --- | --- |
| **(Intercept)** | 0.000 | 0.000 | 0.000 | NA | NA |  |
| **ISO** | 5.217 | 1.443 | 1.464 | 3.563 | 0.000 | *** |
| **MinT** | 9.860 | 3.520 | 3.573 | 2.759 | 0.006 | ** |
| **MeanT** | -6.909 | 4.150 | 4.212 | 1.640 | 0.101 |  |
| **APrec** | -2.191 | 2.335 | 2.370 | 0.925 | 0.355 |  |
| **PrecS** | -1.193 | 1.080 | 1.095 | 1.089 | 0.276 |  |
| **FOR** | -2.099 | 5.333 | 5.412 | 0.388 | 0.698 |  |
| **CROP** | -1.445 | 3.860 | 3.914 | 0.369 | 0.712 |  |
| **SHRUB** | 0.814 | 2.375 | 2.408 | 0.338 | 0.735 |  |
| **GRASS** | -1.903 | 4.014 | 4.075 | 0.467 | 0.641 |  |

1. Models developed for *V. aspis* in Oja Tirón (with climatic, landcover and both variable types)

Climatic

|  | **Estimate** | **Std. Error** | **Adjusted SE** | **z value** | **Pr(>\|z\|)** |  |
| --- | --- | --- | --- | --- | --- | --- |
| **(Intercept)** | 0.000 | 0.000 | 0.000 | NA | NA |  |
| **ISO** | -9.547 | 3.371 | 3.390 | 2.816 | 0.005 | ** |
| **MinT** | -84.915 | 34.736 | 34.927 | 2.431 | 0.015 | * |
| **MeanT** | 79.089 | 34.390 | 34.578 | 2.287 | 0.022 | * |
| **APrec** | 5.570 | 2.410 | 2.423 | 2.299 | 0.022 | * |
| **PrecS** | -1.992 | 2.881 | 2.895 | 0.688 | 0.491 |  |

Landcover

|  | **Estimate** | **Std. Error** | **Adjusted SE** | **z value** | **Pr(>\|z\|)** |  |
| --- | --- | --- | --- | --- | --- | --- |
| **(Intercept)** | 0.000 | 0.000 | 0.000 | NA | NA |  |
| **FOR** | 7.473 | 1.783 | 1.793 | 4.169 | 0.000 | *** |
| **CROP** | -0.532 | 1.027 | 1.033 | 0.515 | 0.607 |  |
| **SHRUB** | -3.188 | 0.856 | 0.861 | 3.703 | 0.000 | *** |
| **GRASS** | 2.128 | 0.830 | 0.835 | 2.549 | 0.011 | * |

All

|  | **Estimate** | **Std. Error** | **Adjusted SE** | **z value** | **Pr(>\|z\|)** |  |
| --- | --- | --- | --- | --- | --- | --- |
| **(Intercept)** | 0.000 | 0.000 | 0.000 | NA | NA |  |
| **ISO** | -8.217 | 3.703 | 3.723 | 2.207 | 0.027 | * |
| **MinT** | -97.454 | 44.656 | 44.901 | 2.170 | 0.030 | * |
| **MeanT** | 92.981 | 44.915 | 45.162 | 2.059 | 0.040 | * |
| **APrec** | 5.718 | 2.976 | 2.992 | 1.911 | 0.056 | . |
| **PrecS** | -1.564 | 2.962 | 2.976 | 0.526 | 0.599 |  |
| **FOR** | 7.016 | 5.559 | 5.589 | 1.255 | 0.209 |  |
| **CROP** | 1.234 | 3.777 | 3.798 | 0.325 | 0.745 |  |
| **SHRUB** | 1.547 | 3.387 | 3.405 | 0.454 | 0.650 |  |
| **GRASS** | -0.937 | 3.263 | 3.281 | 0.286 | 0.775 |  |

1. Models developed for *V. aspis* in Tierras Altas (with climatic, landcover and both variable types)

Climatic

|  | **Estimate** | **Std. Error** | **Adjusted SE** | **z value** | **Pr(>\|z\|)** |  |
| --- | --- | --- | --- | --- | --- | --- |
| **(Intercept)** | 0.000 | 0.000 | 0.000 | NA | NA |  |
| **ISO** | 5.986 | 4.195 | 4.250 | 1.409 | 0.159 |  |
| **MinT** | 17.409 | 13.380 | 13.573 | 1.283 | 0.200 |  |
| **MeanT** | -22.596 | 14.998 | 15.212 | 1.485 | 0.137 |  |
| **APrec** | -7.141 | 2.127 | 2.158 | 3.308 | 0.001 | *** |
| **PrecS** | 2.243 | 0.989 | 1.002 | 2.239 | 0.025 | * |
|  |  |  |  |  |  |  |

Landcover

|  | **Estimate** | **Std. Error** | **Adjusted SE** | **z value** | **Pr(>\|z\|)** |  |
| --- | --- | --- | --- | --- | --- | --- |
| **(Intercept)** | 0.000 | 0.000 | 0.000 | NA | NA |  |
| **FOR** | -1.628 | 1.279 | 1.296 | 1.256 | 0.209 |  |
| **CROP** | -2.769 | 1.291 | 1.310 | 2.113 | 0.035 | * |
| **SHRUB** | -2.665 | 0.894 | 0.906 | 2.942 | 0.003 | ** |
| **GRASS** | 0.974 | 1.150 | 1.167 | 0.835 | 0.404 |  |

All

|  | **Estimate** | **Std. Error** | **Adjusted SE** | **z value** | **Pr(>\|z\|)** |  |
| --- | --- | --- | --- | --- | --- | --- |
| **(Intercept)** | 0.000 | 0.000 | 0.000 | NA | NA |  |
| **ISO** | 9.453 | 6.243 | 6.331 | 1.493 | 0.135 |  |
| **MinT** | 24.626 | 19.131 | 19.442 | 1.267 | 0.205 |  |
| **MeanT** | -31.203 | 21.254 | 21.600 | 1.445 | 0.149 |  |
| **APrec** | -11.602 | 4.413 | 4.473 | 2.594 | 0.010 | ** |
| **PrecS** | 3.397 | 1.623 | 1.645 | 2.065 | 0.039 | * |
| **FOR** | -8.637 | 4.492 | 4.558 | 1.895 | 0.058 | . |
| **CROP** | -8.064 | 4.653 | 4.717 | 1.710 | 0.087 | . |
| **SHRUB** | -4.047 | 2.398 | 2.434 | 1.663 | 0.096 | . |
| **GRASS** | -6.323 | 3.824 | 3.882 | 1.629 | 0.103 |  |

1. Models developed for *V. aspis* in North Iberia (with climatic, landcover and both variable types)

Climatic

|  | **Estimate** | **Std. Error** | **Adjusted SE** | **z value** | **Pr(>\|z\|)** |  |
| --- | --- | --- | --- | --- | --- | --- |
| **(Intercept)** | 0.000 | 0.000 | 0.000 | NA | NA |  |
| **ISO** | 9.348 | 0.782 | 0.784 | 11.929 | < 2e-16 | *** |
| **MinT** | -6.923 | 1.043 | 1.045 | 6.624 | < 2e-16 | *** |
| **MeanT** | 6.007 | 1.193 | 1.195 | 5.027 | 0.000 | *** |
| **APrec** | 1.911 | 0.646 | 0.647 | 2.953 | 0.003 | ** |
| **PrecS** | -1.945 | 0.285 | 0.286 | 6.804 | < 2e-16 | *** |

Landcover

|  | **Estimate** | **Std. Error** | **Adjusted SE** | **z value** | **Pr(>\|z\|)** |  |
| --- | --- | --- | --- | --- | --- | --- |
| **(Intercept)** | 0.000 | 0.000 | 0.000 | NA | NA |  |
| **FOR** | -0.026 | 0.410 | 0.411 | 0.064 | 0.949 |  |
| **CROP** | -0.042 | 0.516 | 0.517 | 0.080 | 0.936 |  |
| **SHRUB** | -0.112 | 0.280 | 0.281 | 0.399 | 0.690 |  |
| **GRASS** | 0.628 | 0.364 | 0.365 | 1.722 | 0.085 | . |

All

|  | **Estimate** | **Std. Error** | **Adjusted SE** | **z value** | **Pr(>\|z\|)** |  |
| --- | --- | --- | --- | --- | --- | --- |
| **(Intercept)** | 0.000 | 0.000 | 0.000 | NA | NA |  |
| **ISO** | 10.012 | 0.843 | 0.845 | 11.852 | < 2e-16 | *** |
| **MinT** | -7.465 | 1.084 | 1.086 | 6.872 | < 2e-16 | *** |
| **MeanT** | 7.156 | 1.273 | 1.275 | 5.612 | < 2e-16 | *** |
| **APrec** | 2.112 | 0.681 | 0.682 | 3.097 | 0.002 | ** |
| **PrecS** | -2.002 | 0.291 | 0.291 | 6.875 | < 2e-16 | *** |
| **FOR** | -0.588 | 0.755 | 0.757 | 0.777 | 0.437 |  |
| **CROP** | -1.588 | 0.978 | 0.980 | 1.620 | 0.105 |  |
| **SHRUB** | -0.555 | 0.456 | 0.456 | 1.216 | 0.224 |  |
| **GRASS** | 0.139 | 0.621 | 0.622 | 0.223 | 0.824 |  |

1. Models developed for *V. latastei* in the High Ebro (with climatic, landcover and both variable types)

Climatic

|  | **Estimate** | **Std. Error** | **Adjusted SE** | **z value** | **Pr(>\|z\|)** |  |
| --- | --- | --- | --- | --- | --- | --- |
| **(Intercept)** | 0.000 | 0.000 | 0.000 | NA | NA |  |
| **ISO** | 3.572 | 1.968 | 1.995 | 1.791 | 0.073 | . |
| **MinT** | -10.872 | 5.231 | 5.302 | 2.051 | 0.040 | * |
| **MeanT** | 5.916 | 6.411 | 6.497 | 0.911 | 0.363 |  |
| **APrec** | -10.198 | 4.791 | 4.853 | 2.101 | 0.036 | * |
| **PrecS** | 1.667 | 1.534 | 1.555 | 1.072 | 0.284 |  |

Landcover

|  | **Estimate** | **Std. Error** | **Adjusted SE** | **z value** | **Pr(>\|z\|)** |  |
| --- | --- | --- | --- | --- | --- | --- |
| **(Intercept)** | 0.000 | 0.000 | 0.000 | NA | NA |  |
| **FOR** | -6.231 | 3.230 | 3.265 | 1.909 | 0.056 | . |
| **CROP** | -2.590 | 2.018 | 2.039 | 1.270 | 0.204 |  |
| **SHRUB** | 0.646 | 1.522 | 1.537 | 0.420 | 0.674 |  |
| **GRASS** | -4.404 | 2.589 | 2.616 | 1.683 | 0.092 | . |

All

|  | **Estimate** | **Std. Error** | **Adjusted SE** | **z value** | **Pr(>\|z\|)** |  |
| --- | --- | --- | --- | --- | --- | --- |
| **(Intercept)** | 0.000 | 0.000 | 0.000 | NA | NA |  |
| **ISO** | 5.090 | 2.484 | 2.516 | 2.023 | 0.043 | * |
| **MinT** | -11.962 | 7.055 | 7.154 | 1.672 | 0.095 | . |
| **MeanT** | 5.859 | 8.548 | 8.666 | 0.676 | 0.499 |  |
| **APrec** | -13.415 | 6.393 | 6.476 | 2.072 | 0.038 | * |
| **PrecS** | 2.543 | 1.604 | 1.626 | 1.564 | 0.118 |  |
| **FOR** | -6.652 | 10.166 | 10.305 | 0.646 | 0.519 |  |
| **CROP** | -1.008 | 6.304 | 6.390 | 0.158 | 0.875 |  |
| **SHRUB** | 1.219 | 3.636 | 3.687 | 0.331 | 0.741 |  |
| **GRASS** | -7.271 | 8.354 | 8.467 | 0.859 | 0.391 |  |

1. Models developed for *V. latastei* in the Oja-Tirón (with climatic, landcover and both variable types)

Climatic

|  | **Estimate** | **Std. Error** | **Adjusted SE** | **z value** | **Pr(>\|z\|)** |  |
| --- | --- | --- | --- | --- | --- | --- |
| **(Intercept)** | 0.000 | 0.000 | 0.000 | NA | NA |  |
| **ISO** | 9.870 | 4.270 | 4.297 | 2.297 | 0.022 | * |
| **MinT** | 124.058 | 182.642 | 183.734 | 0.675 | 0.500 |  |
| **MeanT** | -108.293 | 177.704 | 178.771 | 0.606 | 0.545 |  |
| **APrec** | -9.892 | 4.000 | 4.030 | 2.455 | 0.014 | * |
| **PrecS** | 5.154 | 2.943 | 2.960 | 1.741 | 0.082 | . |

Lancover

|  | **Estimate** | **Std. Error** | **Adjusted SE** | **z value** | **Pr(>\|z\|)** |  |
| --- | --- | --- | --- | --- | --- | --- |
| **(Intercept)** | 0.000 | 0.000 | 0.000 | NA | NA |  |
| **FOR** | -12.630 | 4.952 | 4.988 | 2.532 | 0.011 | * |
| **CROP** | -8.619 | 6.305 | 6.352 | 1.357 | 0.175 |  |
| **SHRUB** | -0.650 | 1.741 | 1.754 | 0.370 | 0.711 |  |
| **GRASS** | -6.344 | 3.251 | 3.274 | 1.938 | 0.053 | . |

All

|  | **Estimate** | **Std. Error** | **Adjusted SE** | **z value** | **Pr(>\|z\|)** |  |
| --- | --- | --- | --- | --- | --- | --- |
| **(Intercept)** | 0.000 | 0.000 | 0.000 | NA | NA |  |
| **ISO** | 23.683 | 25.986 | 26.122 | 0.907 | 0.365 |  |
| **MinT** | 266.394 | 5407.043 | 5451.501 | 0.049 | 0.961 |  |
| **MeanT** | -246.107 | 5280.140 | 5323.562 | 0.046 | 0.963 |  |
| **APrec** | -22.787 | 23.439 | 23.575 | 0.967 | 0.334 |  |
| **PrecS** | 6.462 | 8.617 | 8.670 | 0.745 | 0.456 |  |
| **FOR** | -26.946 | 31.068 | 31.302 | 0.861 | 0.389 |  |
| **CROP** | -17.783 | 16.975 | 17.113 | 1.039 | 0.299 |  |
| **SHRUB** | -4.090 | 10.747 | 10.805 | 0.379 | 0.705 |  |
| **GRASS** | -8.174 | 9.441 | 9.508 | 0.860 | 0.390 |  |

1. Models developed for *V. latastei* in the Tierras Altas (with climatic, landcover and both variable types)

Climatic

|  | **Estimate** | **Std. Error** | **Adjusted SE** | **z value** | **Pr(>\|z\|)** |
| --- | --- | --- | --- | --- | --- |
| **(Intercept)** | 0.000 | 0.000 | 0.000 | NA | NA |
| **ISO** | -2088.800 | 177471.700 | 180268.100 | 0.012 | 0.991 |
| **MinT** | -4497.900 | 398232.100 | 404506.900 | 0.011 | 0.991 |
| **MeanT** | 5686.000 | 501362.700 | 509262.500 | 0.011 | 0.991 |
| **APrec** | 960.300 | 80839.400 | 82113.200 | 0.012 | 0.991 |
| **PrecS** | -320.100 | 27434.300 | 27866.600 | 0.011 | 0.991 |

Landcover

|  | **Estimate** | **Std. Error** | **Adjusted SE** | **z value** | **Pr(>\|z\|)** |
| --- | --- | --- | --- | --- | --- |
| **(Intercept)** | 0.000 | 0.000 | 0.000 | NA | NA |
| **FOR** | -4.110 | 2.671 | 2.712 | 1.516 | 0.130 |
| **CROP** | 0.721 | 2.553 | 2.593 | 0.278 | 0.781 |
| **SHRUB** | -0.157 | 1.208 | 1.226 | 0.128 | 0.898 |
| **GRASS** | -2.312 | 2.137 | 2.170 | 1.066 | 0.287 |

All

|  | **Estimate** | **Std. Error** | **Adjusted SE** | **z value** | **Pr(>\|z\|)** |
| --- | --- | --- | --- | --- | --- |
| **(Intercept)** | 0.000 | 0.000 | 0.000 | NA | NA |
| **ISO** | -847.000 | 169295.900 | 172110.100 | 0.005 | 0.996 |
| **MinT** | -2452.400 | 655987.600 | 666891.800 | 0.004 | 0.997 |
| **MeanT** | 3098.700 | 770573.900 | 783382.800 | 0.004 | 0.997 |
| **APrec** | 509.100 | 105839.100 | 107598.400 | 0.005 | 0.996 |
| **PrecS** | -206.400 | 51079.500 | 51928.600 | 0.004 | 0.997 |
| **FOREST** | -395.300 | 213274.300 | 216819.400 | 0.002 | 0.999 |
| **CROP** | -245.100 | 277655.300 | 282270.600 | 0.001 | 0.999 |
| **SHRUB** | -159.400 | 138702.200 | 141007.700 | 0.001 | 0.999 |
| **GRASS** | -253.000 | 163927.400 | 166652.200 | 0.002 | 0.999 |

1. Models developed for *V. latastei* in North Iberia (with climatic, landcover and both variable types)

Climatic

|  | **Estimate** | **Std. Error** | **Adjusted SE** | **z value** | **Pr(>\|z\|)** |  |
| --- | --- | --- | --- | --- | --- | --- |
| **(Intercept)** | 0.000 | 0.000 | 0.000 | NA | NA |  |
| **ISO** | 6.948 | 1.459 | 1.461 | 4.756 | 0.000 | *** |
| **MinT** | -14.042 | 2.017 | 2.020 | 6.950 | < 2e-16 | *** |
| **MeanT** | 2.861 | 0.788 | 0.789 | 3.627 | 0.000 | *** |
| **APrec** | -19.385 | 2.772 | 2.777 | 6.981 | < 2e-16 | *** |
| **PrecS** | -0.518 | 0.617 | 0.618 | 0.838 | 0.402 |  |

Landcover

|  | **Estimate** | **Std. Error** | **Adjusted SE** | **z value** | **Pr(>\|z\|)** |  |
| --- | --- | --- | --- | --- | --- | --- |
| **(Intercept)** | 0.000 | 0.000 | 0.000 | NA | NA |  |
| **FOR** | -2.444 | 0.350 | 0.351 | 6.974 | <2e-16 | *** |
| **CROP** | 0.779 | 0.321 | 0.321 | 2.424 | 0.015 | * |
| **SHRUB** | 0.534 | 0.269 | 0.270 | 1.978 | 0.048 | * |
| **GRASS** | 0.516 | 0.284 | 0.284 | 1.816 | 0.069 | . |

All

|  | **Estimate** | **Std. Error** | **Adjusted SE** | **z value** | **Pr(>\|z\|)** |  |
| --- | --- | --- | --- | --- | --- | --- |
| **(Intercept)** | 0.000 | 0.000 | 0.000 | NA | NA |  |
| **ISO** | 7.015 | 1.769 | 1.772 | 3.959 | 0.000 | *** |
| **MinT** | -18.907 | 2.991 | 2.996 | 6.311 | < 2e-16 | *** |
| **MeanT** | 2.884 | 1.085 | 1.087 | 2.654 | 0.008 | ** |
| **APrec** | -21.072 | 4.132 | 4.138 | 5.093 | 0.000 | *** |
| **PrecS** | 1.364 | 0.950 | 0.951 | 1.434 | 0.152 |  |
| **FOR** | -3.486 | 1.360 | 1.362 | 2.559 | 0.010 | * |
| **CROP** | 2.143 | 1.185 | 1.187 | 1.805 | 0.071 | . |
| **SHRUB** | 0.772 | 1.014 | 1.016 | 0.760 | 0.447 |  |
| **GRASS** | -1.577 | 1.214 | 1.216 | 1.297 | 0.194 |  |
